# Advanced Ophthalmic Drug Delivery in Homocystinuria Type CblC: A Translational Review of Ophthalmic Pathologies and Therapeutic Opportunities

**DOI:** 10.3390/pharmaceutics18091182

**Published:** 2026-09-19

**Authors:** Selene Cuello-Rodríguez, Martina Sanna, Imelda Lecoeuche, Bruno Sarmento, Francisco J. Otero-Espinar, Victoria Díaz-Tomé

**Affiliations:** 1 Department of Pharmacology, Pharmacy and Pharmaceutical Technology, University of Santiago de Compostela (USC), 15705 Santiago de Compostela, Spain; selene.cuello@rai.usc.es (S.C.-R.); martina.sanna@usc.es (M.S.); imelda.lecoeuche@icloud.com (I.L.); 2 Institute of Materials (iMATUS), University of Santiago de Compostela (USC), 15706 Santiago de Compostela, Spain; 3 Paraquasil Group, University Clinical Hospital, Health Research Institute of Santiago de Compostela (IDIS), 15706 Santiago de Compostela, Spain; 4 Instituto de Investigação e Inovação em Saúde (i3S), Universidade do Porto, 42000-135 Porto, Portugal; bruno.sarmento@i3s.pt

**Keywords:** homocystinuria CblC, hydroxocobalamin, clinical conditions, ophthalmic manifestations, ophthalmic administration, advanced delivery systems

## Abstract

Homocystinuria type CblC is a congenital error in cobalamin metabolism characterized by increased homocysteine and methylmalonic acid levels, together with a reduced methionine availability. This metabolic imbalance leads to multisystemic pathologies, including ophthalmic pathologies that cause damage to the photoreceptors and oculomotor disorders, such as pigmentary retinopathy and nystagmus, respectively. Current commercial treatment (Megamilbedoce^®^) is mainly based on intramuscular hydroxocobalamin administration. Although this approach may improve systemic manifestations, it is ineffective at the ophthalmic level. Given the ophthalmic complications, there is a clear need for new therapeutic strategies capable of significantly reaching the eyeball, improving visual function and patient quality of life. The most convenient and acceptable route for patients is topical–ophthalmic administration. However, treatments administered by this route have significant limitations due to anatomical and physiological ophthalmic barriers. In this context, advanced ophthalmic drug delivery systems have gained increasing interest as strategies to address the low ocular bioavailability of conventional treatments. By increasing ocular residence time, limiting drug loss through tear turnover and nasolacrimal drainage, and improving interaction with ocular tissues, these platforms may enhance local drug availability and therapeutic performance. Nanoparticles, lipid-based carriers, hydrogels, in situ gelling systems, surfactant-based formulations and other multifunctional delivery approaches have shown potential to improve drug retention, tissue penetration and controlled release within the ocular environment. Although reaching posterior ocular tissues through topical administration remains a major pharmacological challenge, these technologies open new possibilities for developing less invasive and more effective treatments for the ocular manifestations of CblC-type homocystinuria.

## 1. Introduction

Vitamin B12 or cobalamin (Cbl) is a water-soluble vitamin obtained through diet. It is present in large quantities in offal such as liver (26–58 µg/100 g), also in smaller amounts in beef, lamb, chicken, eggs and dairy products. This vitamin plays an important role in metabolism, particularly in DNA synthesis, in methylation and mitochondrial metabolism [1]. Consumption below the recommended values (4 µg/day for adults and children and 1.5 µg/day for newborns aged 7 to 11 months [2]) can lead to serious pathologies such as pernicious anemia and cardiac and neurological malformations [3].

Cbl deficiency is a prevalent pathology, whether due to insufficient dietary intake, poor absorption, or enzymatic defects, which can cause diseases such as homocystinuria.

Homocystinuria is the most common autosomal recessive congenital error [4] (the mutated gene is inherited from both parents) of Cbl metabolism, which is involved in the conversion of homocysteine (Hcy) to methionine (Met). This rare hereditary disease affects newborns, infants and adults as well. Homocystinuria can occur in isolation or in association with methylmalonic aciduria (MMA) [5]. MMA is a disease caused by a defect in organic acid metabolism, leading to the accumulation of methylmalonic acid (MA). MMA is caused by a deficiency of enzyme methylmalonyl-CoA mutase (MUT) and Cbl-related metabolic disorders [6].

There are many variants of homocystinuria (CblC, CblD, CblE, CblG, CblX and CblJ) (Table 1), with mutations in the CBS gene or cystathionine beta-synthase (type I homocystinuria), in the MMADHC gene or methylmalonic aciduria protein of the CblC type with homocystinuria (type II homocystinuria), in the MMADHD gene or methylmalonic protein of the CblD type with homocystinuria, in the MTHFR gene or methylene tetrahydrofolate reductase, and in the MTR gene or methionine synthase enzyme (type III homocystinuria), among others. These disorders share an error in the Hcy–Met cycle, leading to elevated Hcy levels and a decrease in Met [7].

**Table 1:** Types of homocystinuria and corresponding mutations [8,9,10].

Type of Homocystinuria	Mutation	Chromosome Involved	Consequence
CblA	Gen MMAA	4q31.1-2	Damage to the synthesis processes of adenosylcobalamin or cobamamide (AdoCbl) causing MMA [8]
CblB	Gen MMAB	12q24.11	
CblC	Gen MMACHC	1p34	Error in the reduction of cobalt present in Cbl and conversion to AdoCbl and methylcobalamin (MeCbl) [8,10]
CblD	Gen MMADHC	2q23	Error in the direction of cobalamin to MUT and methionine synthase (MS) [8,10]
CblE	Gen MTR	5p15.3–p15	Error converting Cbl to MeCbl [8,9]
CblF	Gen LMBRD1	6q13	Defect in the lysosomal release of hydroxocobalamin (HCb) into the cytoplasm [8,10]
CblG	Gen MTR		Defects in MS [8,9]
CblX	Gen HCFC1	Xq28	Inhibit the transcriptional activation of MMACHC [10]
CblJ	Gen ABCD4	14q24	Error in lysosomal release of Cbl into the cytoplasm [10]
Methylmalonyl-CoA epimerase deficiency	Gen MCEE	2p13.3	Can lead to benign subtypes [10]
ADP-formylsuccinyl-CoA synthetase	Gen SUCLA2	13q14.2	

The two main types are classic homocystinuria (CBS enzyme deficiency) and type C, type II or CblC homocystinuria (cobalamin cofactor deficiency) [7]. This review will focus on CblC, whose mutation is located on chromosome 1p34 of the MMACHC gene.

The metabolism of the disease will be discussed, as will its diagnosis and various associated conditions, with an emphasis on ophthalmic conditions such as pigmentary retinopathy and nystagmus. Current commercial treatment focuses solely on alleviating systemic symptoms and is therefore ineffective from an ophthalmic perspective. The ophthalmic route of administration has numerous limitations due to anatomical and physiological barriers in the eye. Consequently, the paper will discuss various advanced systems for the administration of ophthalmic drugs, such as nanoparticles or in situ gelation systems, which have demonstrated their potential to improve drug retention, tissue penetration and controlled release within the ocular environment.

## 2. Biochemistry of Metabolism Hcy and Met

Hcy is a sulfur-containing amino acid obtained from the metabolism of Met. Met is an amino acid derived from both the diet and the catabolism of endogenous proteins and is the only source of Hcy in vertebrates. Numerous reactions occur within the cell, leading to the conversion of Met into Hcy [11].

**Figure 1:**
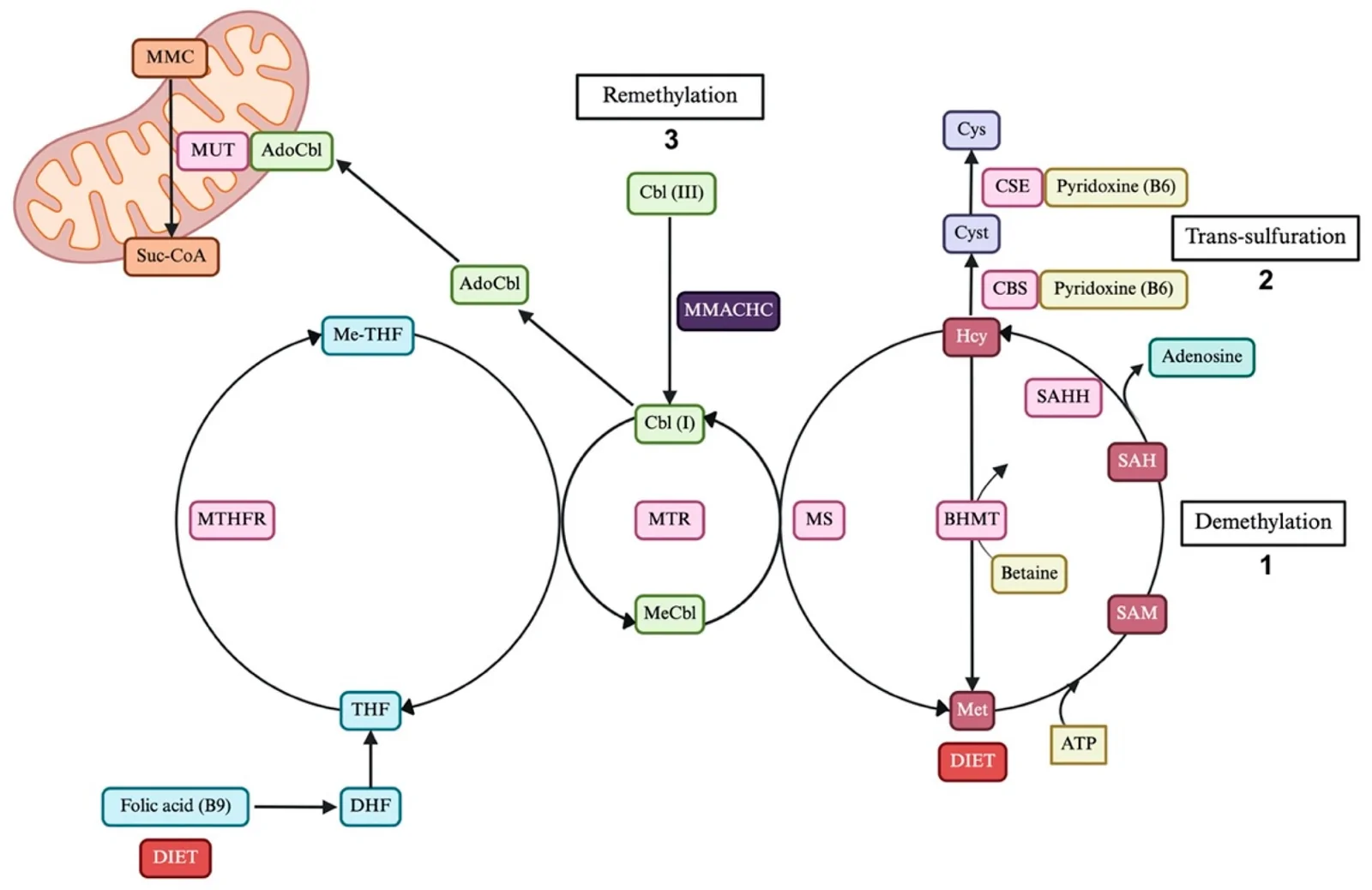
Biochemistry of Hcy and Met metabolism. Metabolic pathways: demethylation pathway (1), transsulfuration pathway (2) and remethylation pathway (3). Abbreviations: AdoCbl, adenosylcobalamin; ATP, adenosine triphosphate; BHMT, betaine-homocysteine methyltransferase; Cbl, cobalamin; DHF, dihydrofolate; Hcy, homocysteine; MeCbl, methylcobalamin; Met, methionine; Me-THF, N5-methyltetrahydrofolate; MMACHC, methylmalonic aciduria type CblC protein with homocystinuria; MMC, methylmalonyl-CoA; MS, methionine mutase; SAH, S-adenosylhomocysteine; SAHH, S-adenosyl-L-homocysteine hydrolase; SAM, S-adenosylmethionine; Suc-CoA, succinyl-CoA; THF, tetrahydrofolate. Created in BioRender. Otero, F. (2026) https://core.local.biorender.dev/api/short-link/p505494.

The Met–Hcy cycle includes three main metabolic pathways (Figure 1): the folate cycle, the choline/betaine cycle and adenosine metabolism.

Metabolic pathway 1, or demethylation pathway: Met, through ATP consumption, is converted into S-adenosylmethionine (SAM), which donates its methyl group and is transformed into S-adenosylhomocysteine (SAH). A reversible reaction converts SAH into Hcy and adenosine, thereby also interacting with adenosine metabolism. This reaction occurs through S-adenosyl-L-homocysteine hydrolase (SAHH). Although this reaction is reversible, the equilibrium is shifted toward SAH formation, such that an increase in Hcy would lead to an increase in SAH, which is an inhibitor of numerous methyltransferase reactions [11,12].

Metabolic pathway 2, or transsulfuration pathway: Hcy is converted into cysteine and taurine through two reactions that are vitamin B6- or pyridoxine-dependent. The first reaction involves the condensation of Hcy with a molecule of serine to form cystathionine, catalyzed by the enzyme CBS [13]. Cystathionine, through the action of the enzyme cystathionine ß-lyase (CSE), yields cysteine and beta-oxobutyrate. This pathway is considered as an elimination pathway for Hcy, which occurs in the liver and kidney. In other organs, the only options for eliminating amino acid would be remethylation to regenerate Met or expulsion outside the cell [11,12].

Metabolic pathway 3, or remethylation pathway: Hcy recovers the methyl group originating from the folate cycle or from choline/betaine metabolism, thereby being converted back into Met. During remethylation, Hcy receives the methyl group from N5-methyltetrahydrofolate (Me-THF), which is formed from 5,10-methylenetetrahydrofolate by methylenetetrahydrofolate reductase (MTHFR) [13]. Although the liver and kidneys participate in the elimination of Hcy through the trans-sulfuration pathway, they also contain betaine-homocysteine methyltransferase (BHMT), which is an alternative form of Hcy remethylation. BHMT with betaine as a cofactor converts Hcy into Met [11].

### 2.1. Connection Between the Remethylation Pathway and Cbl

Mammals possess two Cbl-dependent enzymes, MS and MUT. MS (located in the cytosol) uses MeCbl as a cofactor and Me-THF as a substrate to catalyze the methylation of Hcy to form Met, through the methyltransferase enzyme (MTR). MUT (located in the mitochondria) uses AdoCbl as a cofactor to catalyze the conversion of methylmalonyl-CoA (MMC) into succinyl-Coa (Suc-CoA) [12].

Defective transformation of dietary Cbl into the enzymatically active forms of MeCbl and AdoCbl results in the loss of MS and MUT activities, leading to biochemical abnormalities and systemic pathologies [5,14].

### 2.2. Imbalances in the Met–Hcy Cycle

In the liver, excess Met increases SAM (remethylation pathway), which activates CBS (trans-sulfuration pathway) and inhibits MTHFR (remethylation pathway), leading to the main and irreversible conversion of Hcy into cystathionine. Conversely, if Met levels are low, CBS is not activated, and SAM does not inhibit MTHFR, resolution in Hcy being remethylated primarily back into Met to restore its levels.

If they increase the synthesis of Hcy, its catabolism is not inhibited, and its transport to the extracellular space is increased. This export rate reflects the balance between Hcy synthesis and utilization. This is why extracellular Hcy concentration and particularly plasma Hcy are indicators of enzyme activity and of the availability of the cofactors and substrates involved in its metabolism [11,12,13].

## 3. Biochemistry of Cbl Metabolism

**Figure 2:**
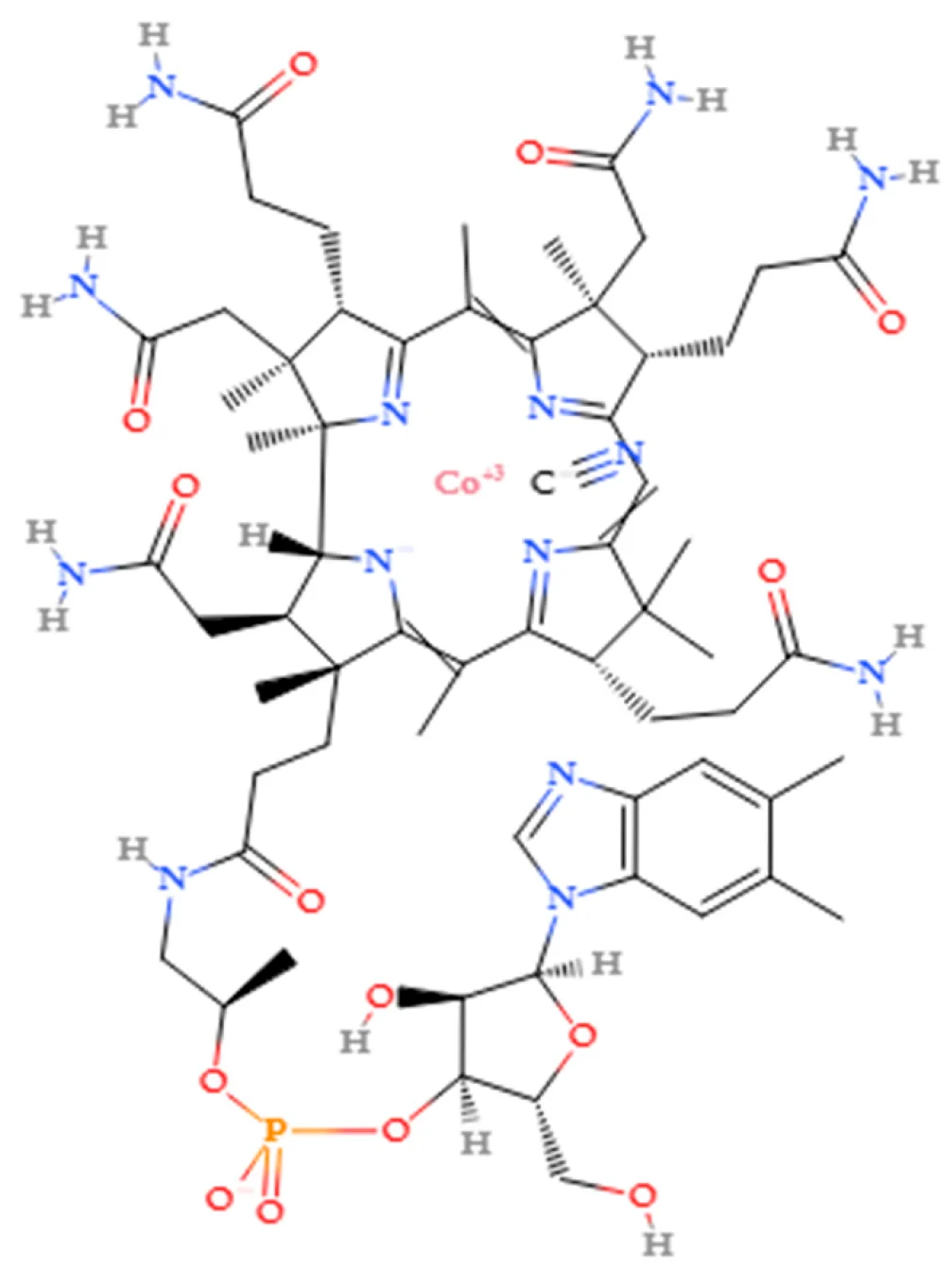
Molecular structure of HCb.

Cbl is a water-soluble vitamin with a complex structure composed of four reduced pyrrole rings forming a macrocyclic ring called the corrin ring (Figure 2) [15]. This macrocyclic ring is chelated by the four nitrogen atoms belonging to the pyrrole rings, displaying a structure like the heme group of hemoglobin (Hb), with cobalt (Co) replacing iron (Fe).

Cbls are similar to nucleic acids because they contain a nucleotide, 5,6-dimethylbenzimidazole, in their structure. The nitrogen in the benzimidazole ring is the fifth covalent coordinate that binds to Co. It also has an a-glycosidic bond in the ribose sugar.

Different anionic ligands can occupy the last Co bond. Depending on the ligand, a specific molecule with different characteristic is obtained. These ligands can be: -CN (cyanocobalamin), -OH (HCb), -NO_2_ (nitrocobalamin), -SO_3_ (sulfitocobalamin), -CH_3_ (methylcobalamin), or -50-deoxy-50-adenisyl (adenosylcobalamin) [16].

**Figure 3:**
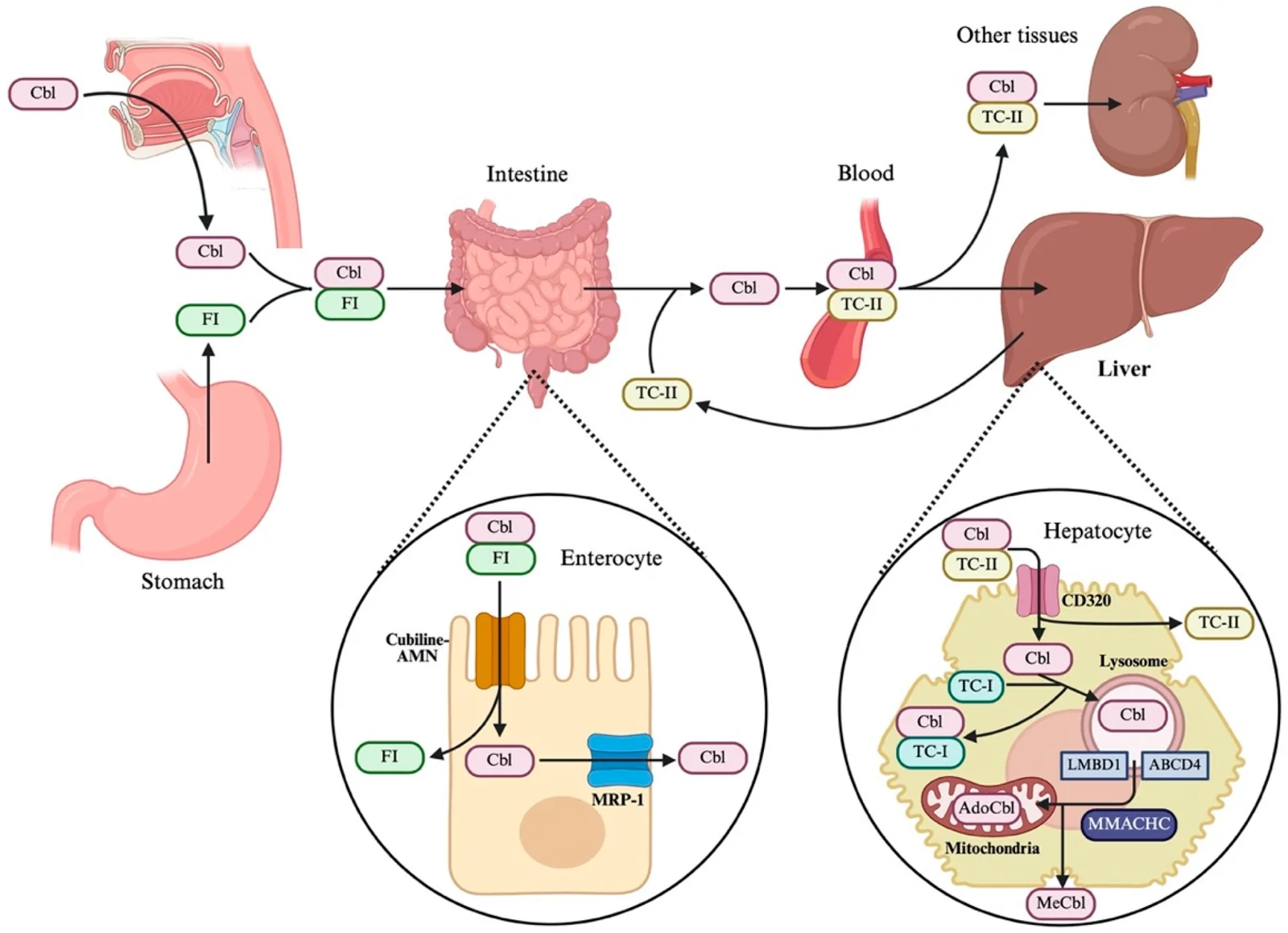
Biochemistry of Cbl metabolism. Abbreviations: ABCD4, subfamily D member 4; ATP, adenosine triphosphate; BHMT, betaine-homocysteine methyltransferase; Cbl, cobalamin; HC, haptocorrin; Hcy, homocysteine; LMBD1, membrane receptor domain 1; Met, methionine; MMACHC, CblC-type methylmalonic aciduria with homocystinuria, MMADHC, CblC-type methylmalonic aciduria with homocysteine; MS, methionine synthase; MUT, methylmalonyl-CoA mutase; TC-I, transcobalamin 1; TC-II, transcobalamin 2. Created in BioRender. Otero, F. (2026) https://core.local.biorender.dev/api/short-link/59i8h27.

Mammals cannot synthesize Cbl, so it must be obtained from the diet through animals’ products (Figure 3) [17]. Cbl obtained from diet is often bound to proteins, forming protein complexes. Upon reaching the stomach, gastric acid (released by the H^+^, K^+^-ATPase pump) [18] and pepsin help break the bonds formed between Cbl and dietary proteins. On the other hand, other proteins are present in both saliva and gastric juice, including haptocorrin (HC), which binds to Cbl in the stomach due to its high affinity for Cbl [15].

Pancreatic trypsin from the pancreatic duct reaches the duodenum through the ampulla of Vaster and, together with other proteases such as chymotrypsin and carboxypeptidase, initiates the degradation of Cbl–protein complexes from the stomach. Once released, Cbl binds to intrinsic factor or IF (a glycoprotein secreted in gastric juice by parietal cells), which undergoes a conformation change that promotes dimerization. This binding is encouraged by the presence of calcium (Ca^2+^), magnesium (Mg^2+^) and bicarbonate (HCO_3_^−^) from pancreatic and biliary secretion [15].

In the distal ileum, Cbl-IF dimers are recognized by a complex receptor protein (cubilin-AMN) [18], which promote absorption by mucosal cells, which slowly release Cbl into the portal blood via the MRP-1 receptor [5,15].

Once in the bloodstream, Cbl binds to a beta-globulin synthesized mainly by the liver, transcobalamin II (TC-II). This complex transports Cbl throughout the body, where it is incorporated into hepatocytes via TCR-mediated endocytosis and degraded [15]. Cbl binds to alpha-transcobalamin or transcobalamin I (TC-I) to be stored, or, if needed, transcobalamin is broken down in the lysosome, and Cbl is released through the CblF and CblJ transports. The transport of free Cbl requires the lopacain-1 receptor, which interacts with the membrane receptor domain 1 (LMBD1) and the subfamily D member 4 binding cassette (ABCD4), two integral membrane proteins.

When Cbl is found in the cytosol, it is processed by MMMACHC, thus entering the Hcy–Met metabolism [5], subsequently transformed into Cbl (II) by MMACHC. Subsequently, the MMADHC transforms Cbl (II) into Cbl (I) and directs Cbl (I) to MS or MUT to complete the cycle [19].

## 4. MMACHC Protein

The gene responsible for CblC-type homocystinuria, MMACHC, is located on chromosome 1p34.1 [20].

The complete MMACHC protein has a molecular weight of 31.7 KDa and contains 282 amino acids [21]. Residues 122-HXXGX-126-154-GG-156 are important for binding to Cbl, as this protein is responsible for catalyzing the decynilation and dealkylation of Cbl to Cbl (II). This subsequently gives rise to the two active forms of Cbl, MeCbl and AdoCbl [20], promoting the passage of Cbl into the cytoplasm.

More than 91 pathogenic mutations have been described in the MMACHC gene, including nonsense mutations, truncation and protein deletion.

The most common is c.271dupA (p.R91KfsX14), a frameshift mutation that accounts for at least 40% of disease-causing alleles. This mutation is presents with severe early-onset disease [22].

The most common nonsense mutations are G147D, which cause vitamin B12-responsive disease and early-onset disease, and c.482 G>A (p.R161Q), where it binds to CNCbl with lower affinity than the wild-type allele (normal and functional form of the enzyme, absent or mutated in the disease) and is associated with late-onset disease [20]. The c.394 C>T (p.R132X) mutation is also associated with late-onset disease [22].

The error in this gene would result in a lack of Cbl (without functional groups), decreasing AdoCbl and MeCbl and leading to deficiencies in MUT and MS.

## 5. Diagnostic Methods

**Figure 4:**
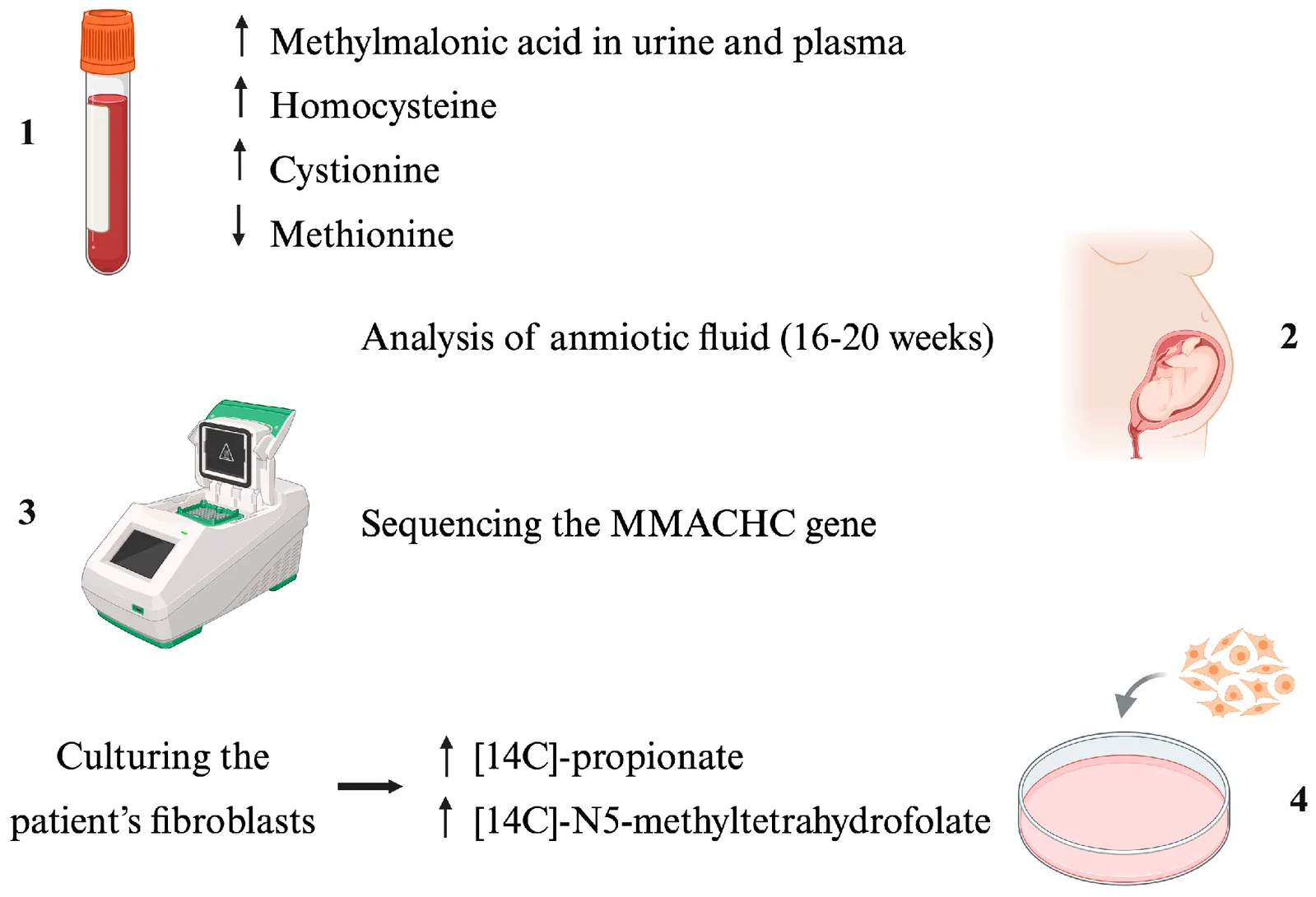
Diagnostics methods. Biochemical parameters measured in blood and urine (1), prenatal analysis of amniotic fluid for early detection (2), molecular diagnosis using PCR (3), and supplementary investigations involving the culture of the patient’s fibroblasts (4). Created in BioRender. Otero, F. (2026) https://core.local.biorender.dev/api/short-link/ro2ta6k.

Diagnosis can be complicated because metabolic acidosis or hyperammonemia may not be detected during neonatal screening, so patients are not usually referred to specialists in metabolic diseases. Due to the rapid progression of the disease, early diagnosis is the key [22] (Figure 4).

### 5.1. Biochemical Parameters

If this disease is suspected, a blood test should be performed, including serum MA, total plasma Hcy, plasma amino acids, acylcarnitine profile (an increase in propionylcarnitine would be observed), and organic acids in urine [12]. An increase in MA, Hcy and cystionine and a decrease in Met is an indication of the disease. Ma, 2-methylcitrate and propionylcarnitine are indicators of AdoCbl and MUT production. Plasma Hcy, plasma Met and cystionine are indicators of MeCbl and MS production [22].

After venipuncture, most Hcy is bound to proteins. After blood collection, the plasma must be deproteinized because free Hcy rapidly binds to proteins in vitro. The problem with using free Hcy as a marker of the disease is that it is only detectable when total plasma Hcy levels exceed 60 µmol/L, so it is preferable to analyze the plasma Hcy level [13].

An increase in urine or plasma MA confirms a Cbl deficiency. In addition to differentiating metabolic or absorption problems with this vitamin from a nutritional deficiency, Cbl or folate is administrated enterally [13].

### 5.2. Molecular Diagnosis

Molecular diagnosis is performed by sequencing the MMACHC gene using polymerase chain reaction (PCR) [23]. This reaction amplifies the coding of introns and exons. Once the PCR is performed, the products obtained are analyzed on an agarose gel [24].

Another option for gene sequencing is PCR-HRM, which includes PCR reaction, amplicon melting and gene scanning analysis. This option consists of the high-throughput real-time fluorescence quantitative PCR system [25].

### 5.3. Prenatal Diagnosis

Prenatal testing consists of a series of tests on the amniotic fluid of pregnant women between 16 and 20 weeks. A karyotype analysis, DNA extraction and metabolite analysis are performed. The latter determines the concentration of Hcy (normal range between 4–10 µmol/L), organic acid (normal range MMA 0.00–1.00) and MCA (monochloroacetic acid 0.00–0.50 mmol/mol) and acylcarnitine (appropriate range C3 0.3–4.00 µmol/L and C3/C2 0.05–0.25) [26].

### 5.4. Complementary Studies

The complementary diagnostic study for this disease consists of culturing fibroblasts from a patient, obtained from skin biopsies, with fibroblast cell lines from a defined complementation group [22]. Two molecules, [14C]-propionate and [14C]-N5-methyltetrahydrofolate, are incorporated into the patient’s fibroblast culture; if the patient does not have this disease, the two molecules would produce amino acids, which in turn would form cellular macromolecules. If, on the other hand, the patient has this disease, the levels of [14C]-propionate (14¦21% above normal) and [14C]-N5-methyltetrahydrofolate (28–39% above normal) would increase [5].

## 6. Clinical Conditions

CblC homocystinuria is a disease that presents systemic symptoms at different stages of life. Depending on age, symptoms can be classified according to the age of onset, with the disease being prenatal, infantile, or microcephaly, or early-onset CblC and adult or late-onset CblC.

Prenatal: the disease can manifest early in the womb, causing growth retardation, facial dysmorphic features, microcephaly or congenital heart disease [12,22].

Infantile: this is known as early onset CblC disease, characterized by feeding difficulties, hypotonia, neurological impairment, hematological abnormalities, and macular changes, and hemolytic uremic syndrome may occur [12,22].

Non-infantile or adult: this is known as late-onset CblC disease (after 4 years of age), in which neuropsychiatric symptoms, progressive encephalopathy, pigmentary retinopathy, spinal cord degeneration, and thromboembolic complications, among others, may occur [12,22].

Below, each pathology caused by CblC is defined, and a series of clinical cases related to each one is reported, where all patients suffered from CblC or CblC associated with MMA.

### 6.1. Microcephaly and Hydrocephalus

Microcephaly is diagnosed when the occipitofrontal head circumference is 2 standard deviations below the expected average for the given percentile, age, sex and population. It is a chronic condition associated with intellectual disability, developmental delay, epilepsy and cerebral palsy, among other pathologies [27,28]. Hydrocephalus is an abnormal accumulation of cerebrospinal fluid (CSF) that changes in intracranial pressure may accompany [29]. Newborns with hydrocephalus may have an increased head circumference, while children often have elevated intracranial pressure [30]. These two pathologies are prevalent in CblC, either as the main symptom (in the prenatal and infant stages) or as an associated feature [22,31,32,33].

### 6.2. Acidosis, Ketonuria and Hyperammonemia

Acidosis is characterized by an increase in hydrogen plasma ion (H^+^) or decrease in plasma HCO_3_^−^ [34]. In CblC, plasma HCO_3_^−^ is decreased (18.5 mmol/L) compared to the normal range (21–28 mmol/L) [35]. Ketonuria is a disorder characterized by high concentration of ketone bodies in the urine (acetoacetate or beta-hydroxybutyrate) [36]. Hyperammonemia is an elevation of the concentration of ammonium (NH_4_^+^) in plasma, which can lead to coma and endanger the brain [37].

Several cases have been reported at the clinic in which patients with CblC also had these associated conditions [32,38,39,40].

### 6.3. Congenital Heart Disease

Severe cases of CblC may present with structural heart defects, including muscular ventricular septal defect, left ventricular non-compaction secundum atrial septal defect, cor pulmonal, mitral valve prolapse with mild mitral regurgitation, dysplastic pulmonary valve without pulmonary stenosis, and right heart failure secondary to pulmonary embolism [41,42].

### 6.4. Megaloblastic Anemia

Megaloblastic anemia is a condition caused by defects in the DNA synthesis of hematopoietic precursors. This causes an error in erythropoiesis and intramedullary hemolysis. This anemia belongs to the megaloblastic anemia group, which is characterized by a high mean corpuscular volume (>100 fL). One of the most common causes is Cbl deficiency, which can result from numerous factors, including CblC [43,44].

### 6.5. Pulmonary Hypertension

Pulmonary hypertension is a condition characterized by an increase in pulmonary artery pressure above 25 mmHg. This leads to right ventricular failure and premature death [45].

Several cases have been reported at the clinic in which patients with CblC also had these associated conditions [46,47,48].

### 6.6. Renal Thrombotic Microangiopathy (RTM)

RTM is a pathological lesion present in multiple disease, triggered by vascular endothelial injury. RTM lesions are often accompanied by microangiopathic hemolytic anemia, thrombocytopenia, and ischemic injury to terminal organs [49]. In addition, the ischemia and inflammation produced can trigger multiple organ failures [50].

Several cases have been reported at the clinic in which patients with CblC also had these associated conditions [47,51,52,53,54,55,56].

### 6.7. Hemolytic Uremic Syndrome

Hemolytic uremic syndrome is a set of pathologies, including autoimmune hemolytic anemia, thrombocytopenia and renal failure, resulting in RTM. This type of syndrome is associated with genetic mutations or antibody alterations [47,57,58].

### 6.8. Neurologic and Neuropsychiatric Manifestations

The physiology of neurological pathologies observed in CblC is unclear. Research has linked elevated Hcy levels with decreased cognitive performance in adults, due to stimulation of the N-methyl-D-aspartate (NMDA) receptor and damage to the vascular endothelium [33,59]. On the other hand, it is possible that elevated plasma MA levels are associated with growth retardation [32,60]. Over the years, cases of depression, psychosis, hallucinations, and behavioral disorders have been reported in this disease [12,61,62,63,64].

## 7. Ophthalmic Manifestations

**Figure 5:**
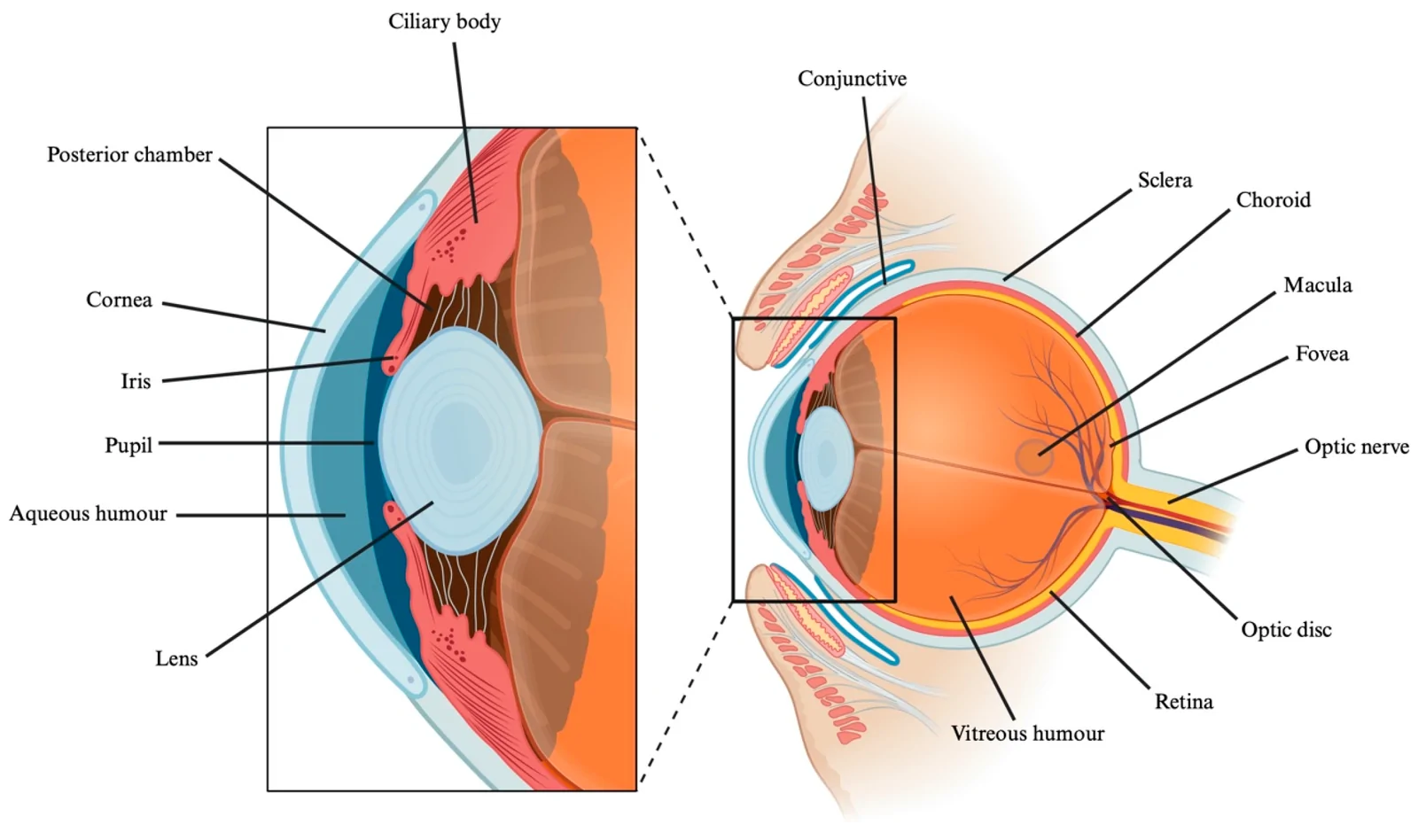
Anatomical parts of the eye including anterior and posterior segments. Created in BioRender. Otero, F. (2026) https://core.local.biorender.dev/api/short-link/35mad2p.

The eye consists of a sensory pathway (the optic tract), a receptor (the retina), and the primary cerebral cortex. This structure is located in the orbital cavity, which contains adipose tissue that provides protection and cushioning. This sensory organ is responsible for detecting light and converting it into nerve impulses. The eye is divided mainly into two segments: the anterior and posterior segments (Figure 5). The anterior segment consists of the limbus, cornea, Schlemm’s canal, trabecular meshwork, anterior and posterior chambers, lens, iris, ciliary body, and zonules. The posterior segment consists of the retina, vitreous humor, sclera, choroid, and optic nerve [65].

The outermost layer, composed of the sclera and cornea, serves to maintain the spherical shape of the eye. The cornea is highly innervated, avascular and transparent. It is composed of the epithelium (the outermost layer, which is lipophilic and has tight junctions), the stroma (a hydrophilic layer), Dua’s layer, Bowman’s membrane (composed mainly of collagen), Descemet’s membrane, and endothelium (the innermost layer, which is lipophilic, provides proper hydration to the cornea, regulates its thickness, and thus ensures its transparency). The sclera is the opaque outer layer of the eye that extends from the corneal limbus to the optic nerve. Located between the sclera and the retinal pigment epithelium (RPE) is the choroid. The choroid is a highly vascularized layer that extends from the ora serrata to the optic nerve and is responsible for supplying the nutrients and oxygen needed by the retina [65]. Covering the sclera is the conjunctiva, an epithelial layer exposed to the outside and the eyelids, whose function is to facilitate lubrication of the eye and adhesion of the tear film [66].

The middle layer contains the main blood supply to the eye, as well as the iris and pupil. Between the iris and the vitreous body is the crystalline lens, the eye’s biconvex converging lens. It is almost transparent and gray in color, and its function is to focus on distant and near objects [67].

The innermost layer is composed of the retina. The retina is a transparent tissue composed of multiple layers, which has an optical part and non-sensory part, divided by the ora serrata (anterior limit of the retina). The optical part is formed by the RPE and the cerebral layer, which provides the neurosensory layer with nutrition and removal of metabolic waste products. The retina contains photoreceptors, which are cones and rods (first neuron), bipolar cells (second neuron), and multipolar ganglion cells (third neuron), in addition to horizontal and amacrine cells [65,66].

The aqueous humor present in the anterior and posterior chambers is a colorless solution composed of amino acids, carbohydrates and oxygen, among other substances. Its purpose is to preserve transparency, maintain adequate intraocular pressure (IOP), and provide nutrition and oxygen while removing metabolic waste products [65,68]. The vitreous humor is a dense, transparent gel located at the back of the eyeball. It is composed mainly of water, along with multiple ions such as sodium. It is responsible for supporting the retina and maintaining its transparency, allowing light-beams to pass through it [65,69].

The eye conditions associated with CblC are high myopia, nystagmus, optic atrophy, strabismus, pigmentary retinopathy, and retinal degeneration with maculopathy. These symptoms are directly related to the age of onset of the disease [70]. Currently, the mechanism or pathway involved in the development of these conditions in patients with CblC has not been described in detail.

### 7.1. High Myopia

High myopia is defined as exceeding 5 diopters. This condition is rare and usually accompanied by an ocular refractive error [71].

According to studies, it is a common refractive error in CblC [72]. Gerth et al. report that this refractive pathology is the most common among the patients studied, including hyperopia. One patient in the late-onset group presented with myopia and changes in the fundus [73]. Nguyen et al. report the case of a young man with early-onset CblC who developed myopia along with other vision abnormalities [74].

### 7.2. Retinitis Pigmentosa

Retinitis pigmentosa is a retinal dystrophy caused by the gradual loss of photoreceptors (rods and cones) with retinal pigment deposits in the fundus. It usually begins with night blindness, followed by progressive loss of peripheral vision and eventually total blindness [75]. As the disease progresses, other associated eye conditions may develop, such as cataracts or macular degeneration [76].

According to various studies, between 35% and 67% of patients with CblC had retinitis pigmentosa [72,73,77]. In addition, Gizicki et al. reported in their study that all patients who had retinal pigmentary changes also had macular abnormalities [78]. In the study by Bacci et al. the patients underwent a routine electroretinogram, which showed a 50% reduction in the scotopic wave and a delay in the photopic wave, indicating a reduction in photoreceptors and bipolar cells [79].

### 7.3. Maculopathy

Maculopathy develops around 3.5 months of age. It begins with pigmented ocular changes progressing to bull’s-eye maculopathy, presenting a hypopigmented perimacular area surrounded by a hyperpigmented ring. It usually progresses toward the periphery of the retina and can cause optic nerve atrophy [80].

This ophthalmic defect is very common among patient with CblC, presenting macular abnormalities from an early age and even leading to macular pseudocolobomas [72,79]. Brooks et al. report that 18 of the 25 patients in their study had this ophthalmic pathology [80]. Several studies show that between 60% and 70% of patients in the early-onset group had this pathology [73,78] and may suffer from other associated ophthalmic pathologies [77,81]. On the other hand, Collison et al. reported an exceptional case of a patient with late-onset disease who had this condition and presented with bull’s-eye macular lesions in both eyes [82].

### 7.4. Optic Atrophy

Optic atrophy is a condition in which the optic nerve narrows due to degeneration of the axons of the retinal ganglion cells. In advanced stages, it may be accompanied by a pallor of the optic disc due to degeneration of the capillaries [81].

According to data collected by Bonafede et al., three of the 11 patients in the study presented with pallor (pallor of the optic disc) or progressive optic nerve atrophy [72]. Gizicki et al. report that six of the 10 patients in the early-onset group of the study had this condition [78]. Gerth et al. found that three of the patients in the early-onset group of the study had this optic nerve atrophy [73]. According to Kalantari et al., this pathology was the most prevalent in the patients in their study, affecting 13% of the patients in the study [83]. According to Weisfed-Adams et al., this pathology is less common in the patients in the study, with only 16 of the 62 patients in the study with early-onset disease presenting it [77].

### 7.5. Nystagmus

The nystagmus is an eye movement disorder characterized by abnormal involuntary rhythmic oscillations of one or both eyes. It can be isolated disorder or associated with other ophthalmic disorders, such as achromatopsia or aniridia, or form part of a multisystemic disorder such as albinism [84].

Several studies have observed numerous cases of nystagmus among study patients, ranging from 54% to 80% [72,78,79,80], some of whom had other associated conditions such as maculopathy [77] and neurological disorders [74]. On the other hand, Gerth et al. report the case of a patient with late-onset disease who presents this condition [73].

### 7.6. Strabismus

Strabismus is an eye condition prevalent in pediatrics [85], where both eyes are not aligned correctly and point in different directions [86]. In addition to being a medical condition, it also causes social and psychiatric problems [85].

According to Weisfeld-Adams et al., this condition is less common in the patients studied, occurring in only 14 of the 62 patients with early-onset disease [77]. Gizicki et al., on the other hand, report that five of the 12 patients in the early-onset group of the disease studied had this condition [78].

## 8. Therapeutic Strategies

The usual treatment for CblC homocystinuria consists of intramuscular (IM) HCb as an alternative to CN-Cbl, due to improved results. This can be seen in a greater decrease in MA in urine and a decrease in plasma Hcy [87]. Furthermore, according to previous studies, it has been observed that the mutated MMACHC gene has a greater affinity for HCb than for CN-Cbl, in addition to presenting a decrease in intracellular reactive oxygen species (ROS) levels [22].

It is common to initiate prenatal therapy in pregnant women with this disease, through IM administration of HCb, with doses of between 1 and 10 mg per day for 2–3 per week, starting in the 15th-week of gestation [88].

### 8.1. Hydroxocobalamin

In the treatment of CblC homocystinuria, an initial dose of 1 mg of HCb administered daily by IM (usual route), intravenous (IV), or subcutaneous (SC) injection is recommended (treatment is usually administered by IM) to reduce Hcy and MA levels and increase Met levels [22,88,89]. Once long-term treatment has been established, each case should be studied individually.

Studies have shown that an increase in daily HCb improves the parameters indicative of the disease, Hcy and MA. Daily doses of 25 mg of HCb improved hematological abnormalities and neurological manifestations [22], in addition to metabolically controlling the disease [5]. Scalais et al. corroborate that doubling the dose of HCb significantly improves the symptoms caused by CblC homocystinuria. Their study reports the case of three pediatric patients with CblC homocystinuria who were administered 2.50 mg/kg/day of HCb as treatment. One of them received double the dose (5.50 mg/kg/day) due to a staff error. Fortunately, the patient did not suffer any harm, and the symptoms caused by the disease improved [90].

Bartholomew et al. conducted a 19-day study in infants to assess the necessary frequency of IM injections of HCb. The study found that 24 h after injection, serum Cbl levels in adults increased approximately 20-fold and decreased to almost zero after 19 days. The half-life of HCb was found to be 24 h in adults and 62 h in children. No significant increases in total plasma Hcy levels were recorded during the study period [91].

### 8.2. Betaine

Betaine is used to reduce Hcy levels and normalize Met levels, in synergy with HCb [22]. It is normally administered orally at a dose of 250 mg/kg daily [5].

Bartholomew et al. conducted a study evaluating betaine supplementation in conjunction with HCb. The study found that betaine supplementation helped to decrease Hcy levels and increase Met levels in a synergistic effect [91].

### 8.3. Folate, Met and Carnitine

The involvement of folate in CblC homocystinuria has not yet been demonstrated, but it is believed to promote the conversion of Hcy to Met [22]. The efficacy of Met and carnitine supplementation in CblC homocystinuria has also not been demonstrated. Still, because the disease decreases Met and L-carnitine levels, supplementation with these substances is considered [22,88,89,92].

Following is a summary table (Table 2) of the progress, limitations and future perspective of the last five years regarding CblC homocystinuria and its associated pathologies.

**Table 2:** Summary table of the progress, limitations and future perspective of the last five years regarding CblC homocystinuria and its associated pathologies.

	Recent Advances	Limitations	Current Solutions or Future Perspectives	
Molecular mechanism	Further insight has been gained into the role of MMACHC in the intracellular processing of the different forms of Cbl.	The high functional heterogeneity of MMACHC variants makes it difficult to predict phenotype and therapeutic response.	Functional and structural characterization of variants and development of therapeutic strategies tailored to each molecular defect.	[5,20]
Prenatal diagnosis	Measurement of Hcy in amniotic fluid, combined with other metabolic biomarkers.	Requires identification of familial pathogenic variants and access to specialized infrastructure for molecular diagnosis.	Combination of biochemical and molecular diagnosis in at-risk pregnancies.	[26,39]
Clinical and genetic heterogeneity	Expansion of the genotypic and phenotypic spectrum of combined MA and CblC.	Marked variability in age at onset, disease severity, and affected organs.	Genetic and clinical stratification of patients and individualized follow-up according to genotype, phenotype, and biomarkers.	[5,32]
Delayed diagnosis in adults	The adult-onset phenotype has been better characterized and may include neurological, psychiatric, renal, thromboembolic, and ophthalmic manifestations.	The multisystemic and nonspecific presentation may lead to diagnostic delays.	Greater clinical awareness, early measurement of total Hcy, and inclusion of MMACHC and related genes in molecular diagnostic panels.	[13,83]
Incomplete metabolic treatment	Parenteral HCb remains the cornerstone of treatment, with increasing recognition of the need to individualize dose and administration frequency.	There is no universally optimal treatment regimen. Control of MMA, Hcy, and Met does not guarantee prevention of all complications.	Early treatment initiation, personalization of HCb dosing according to biochemical and clinical response, and development of strategies targeting specific pathogenic mechanisms.	[5,83]
Neuropsychiatric manifestations	The broad spectrum of neurocognitive, behavioral, and psychiatric abnormalities has been better characterized.	Metabolic control does not always reverse established neurological damage, and some abnormalities may persist or progress.	Early diagnosis and treatment, periodic neuropsychological assessment, and multidisciplinary management.	[5,74]
Cardiovascular complications	Different patterns of cardiovascular involvement have been identified, including alterations in ventricular function.	Cardiovascular involvement may remain subclinical and is not always included in routine follow-up.	Periodic cardiovascular screening and monitoring, including ECG and echocardiography in at-risk patients.	[41]
Renal involvement and RTM	Recognition of CblC in the differential diagnosis of patients with RTM and unexplained renal impairment has increased.	Predominantly renal forms may be mistaken for other causes of RTM.	Measurement of Hcy and MMA in unexplained RTM and inclusion of MMACHC in relevant molecular diagnostic panels.	[49,83]
Ophthalmic involvement	Retinopathy, maculopathy, nystagmus, optic atrophy, and visual impairment associated with CblC have been better characterized.	Ophthalmic abnormalities may progress despite early diagnosis and systemic HCb treatment. Currently, no specific therapy is available for the treatment of these manifestations.	Early and longitudinal ophthalmic follow-up and future development of strategies specifically targeting ocular tissues to complement systemic metabolic treatment.	[5,83]

## 9. Improve Parenteral Treatment for the Systemic Diseases

This type of administration route includes IM, SC and IV administration. It has several advantages, including avoiding first-pass metabolism in the liver, improving bioavailability and enabling more reliable dosing. It also allows for more predictable pharmacodynamic and pharmacokinetic profiles [93].

IV administration consists of administering drugs directly into the venous bloodstream. It allows the drug to have an immediate effect, offering the possibility of controlling the rate of distribution and administering large doses quickly. It also allows for continuous administration of medication. The main problem with this type of administration is that the drug must be administered in an outpatient or hospital setting and under controlled conditions, and it is very difficult to reverse the administration [94].

SC administration consists of administering drugs directly into the subcutaneous tissue, which is located beneath the dermis and above the muscle. It allows for self-administration, offers patients greater mobility and comfort, provides an alternative for patients with poor venous access and can be administered at home, unlike intravenous administration [95].

IM administration involves injecting drugs directly into selected muscle, usually large muscle with good vascularity (gluteal, deltoid and lateral biceps). Administration via this route allows the drug to quickly enter systemic circulation, avoiding the first-pass effect of the liver. On the other hand, it is a very painful route that normally requires healthcare personnel for administration [96].

The commercial drug used for this condition is Megamilbedoce^®^ (Aristo Pharma Iberia, S.L.) and is based on an injectable solution of HCb (the hydroxylated active form of Cbl). This drug is administered parenterally, as mentioned above, preferably IM. Megamilbedoce^®^ is indicated for the treatment of systemic pathologies associated with CblC homocystinuria, such as pernicious anemia, with the aim of increasing patient survival and improving quality of life [97]. In the case of ocular complications, this treatment is not effective due to the impossibility of a significant amount reaching the eyeball.

Intramuscular systemic administration results in low ocular bioavailability compared with ophthalmic routes, whilst also requiring high systemic doses, increasing the risk of adverse effects. As reported in the literature, systemic administration results in low ocular bioavailability, which is primarily limited by the blood–aqueous and blood–retinal barriers [98,99].

For systemically administered drugs to reach the ocular tissues, they must remain in the bloodstream, avoid systemic elimination and subsequently cross the selective ocular barriers mentioned above [99]. The permeability of these barriers depends greatly on the properties of the drug; thus, lipophilic drugs cross the ocular barriers more easily than hydrophilic ones [98], whilst HCb is a highly hydrophilic drug.

## 10. Other Administration Routes and Treatment

### 10.1. Oral Administration

The oral route of administration is preferred over other routes of drug administration due to its numerous advantages. These include safety, good compliance and adherence by the patient, and painless administration [93].

The disadvantages of this route include the difficulty of delivering drugs to the ocular targets, the acidic conditions of the stomach or basic conditions of the small and large intestine, which are decisive depending on the characteristics of the drug, possible systemic adverse effects, the first-pass effect in the liver, and possible metabolism in the intestine [100]. This series of drawbacks must be considered when choosing the most appropriate system for the drug to be administered.

Currently, new delivery systems are being developed for administration via this route, allowing the drug release profile to be modified and the formulation to be adapted to the characteristics of the drug, such as mucoadhesive systems or gastric floaters [101,102,103]. PH-dependent systems such as hydrogels, nanoparticles or microspheres that contain a polymer in their structure that responds to changes in pH can also be used [104,105,106].

Bartholomew et al. orally administered a 1 mg dose of HCb to two adult volunteers. The initial dose showed an almost twofold increase in serum Cbl levels at 4 h. On the other hand, when administered three times a day by this route for one week, no significant increase in serum Cbl levels was observed. In both cases, serum Cbl levels were compared with the serum levels of the control subjects [91].

This route of administration could be interesting for treating type C homocystinuria, as it has already been observed that drugs used to treat systemic pathologies can also improve certain ophthalmic diseases. This is the case with Rebamipide, which is mainly used to treat gastric ulcers. In turn, it increases certain molecules very similar to mucin and the number of conjunctival goblet cells on the ocular surface, proving useful in the treatment of dry eye disease (DED). Oral lactoferrin administered for one month is also used to treat this disease. Lactoferrin is secreted by the lacrimal glands as a protector against cell death caused by oxidative stress [107].

### 10.2. Topical Skin Administration

#### 10.2.1. Transdermal Drug Delivery System (TDDS)

Transdermal drug delivery systems (TDDSs) are controlled drug delivery systems in which the drug is administered through the skin to achieve controlled drug delivery. Administration is non-invasive and well tolerated by patients, comfortable, reduces systemic adverse effects, and presents less fluctuation in plasma drug concentration compared to other systems. In addition, it avoids the first-pass effect in the liver, prolonging the effect of the drug in the body. On the other hand, the stratum corneum of the skin acts as a barrier, limiting transdermal permeation. However, nanotechnology can improve this limitation due to the small size that allows it to pass through cell junctions [108]. Other limitations are the difficulty of accurately administering doses, as it would be necessary to measure blood and tissue levels, and skin irritation. Skin irritation could be reduced by using a formulation where the chosen vehicle has a pH range between 5.4 and 5.9 [109,110]. In other words, if we have an acidic drug, it can be encapsulated in nanoparticles, whose final nanosuspension has the appropriate pH. Considering that this system would be suitable for the systemic symptoms of CblC, similar systemic systems have now been developed for transcorneal administration. Microneedles are systems that penetrate the cornea, improving the ocular bioavailability of drugs and being very useful in the treatment of CblC-type homocystinuria. These systems have the disadvantage of damaging the anterior elastic and stromal layers, causing pain during administration, and altering corneal transmittance. Their usefulness and efficacy have been described in abnormal neurovascular diseases such as diabetic retinopathy and age-related macular degeneration (AMD) through the administration of anti-VEGF agents using microneedles [111,112]. Taking advantage of recent research on the application of this type of system in other ophthalmic diseases, it would be interesting to implement it in the treatment of CblC-type homocystinuria.

#### 10.2.2. Implantable Drug Delivery Systems (IDDS_S_)

Implantable drug delivery systems (IDDSs) are surgically implanted devices that serve as reservoirs or depots for prolonged drug delivery, ranging from simple implants to osmotic pumps or microchips [108]. These systems have the advantage of maintaining controlled drug delivery over time, unlike other routes such as oral and IV. This reduces toxicity problems due to peak concentrations at the time of administration and ineffectiveness associated with decreasing concentrations [113].

The small size of IDDSs facilitates their implantation and reduces the discomfort of their administration. In addition, these IDDSs are made from biodegradable materials, eliminating the need to remove them after treatment. Some models offer remote control that allows for precise and controlled drug dosing, as well as monitoring the patient’s condition. The main disadvantages of these systems are the risk of infection or tissue damage due to their surgical implantation, the limited drug load, the possible failure of remote-controlled devices, which requires removal of the implant and administration of a new one, and the need for constant monitoring of the implant’s functioning [114].

There are numerous studies describing the development of ocular implants using different techniques to treat diseases of the posterior segment. Rodrigues do Silva et al. and Bendicho-Lavilla et al. have developed biodegradable ocular implants made of polyurethane and PLGA, respectively, containing dexamethasone for the treatment of uveitis and retinal diseases [115,116]. Ophthalmic implants such as Ozurdex^®^ (AbbVie, Illinois, U.S.A), an intravitreal dexamethasone implant [117], and Iluvien^®^ (Alimera Sciences), an intravitreal fluocinolone acetonide implant [118], are currently available on the market.

### 10.3. Ophthalmic Administration

**Figure 6:**
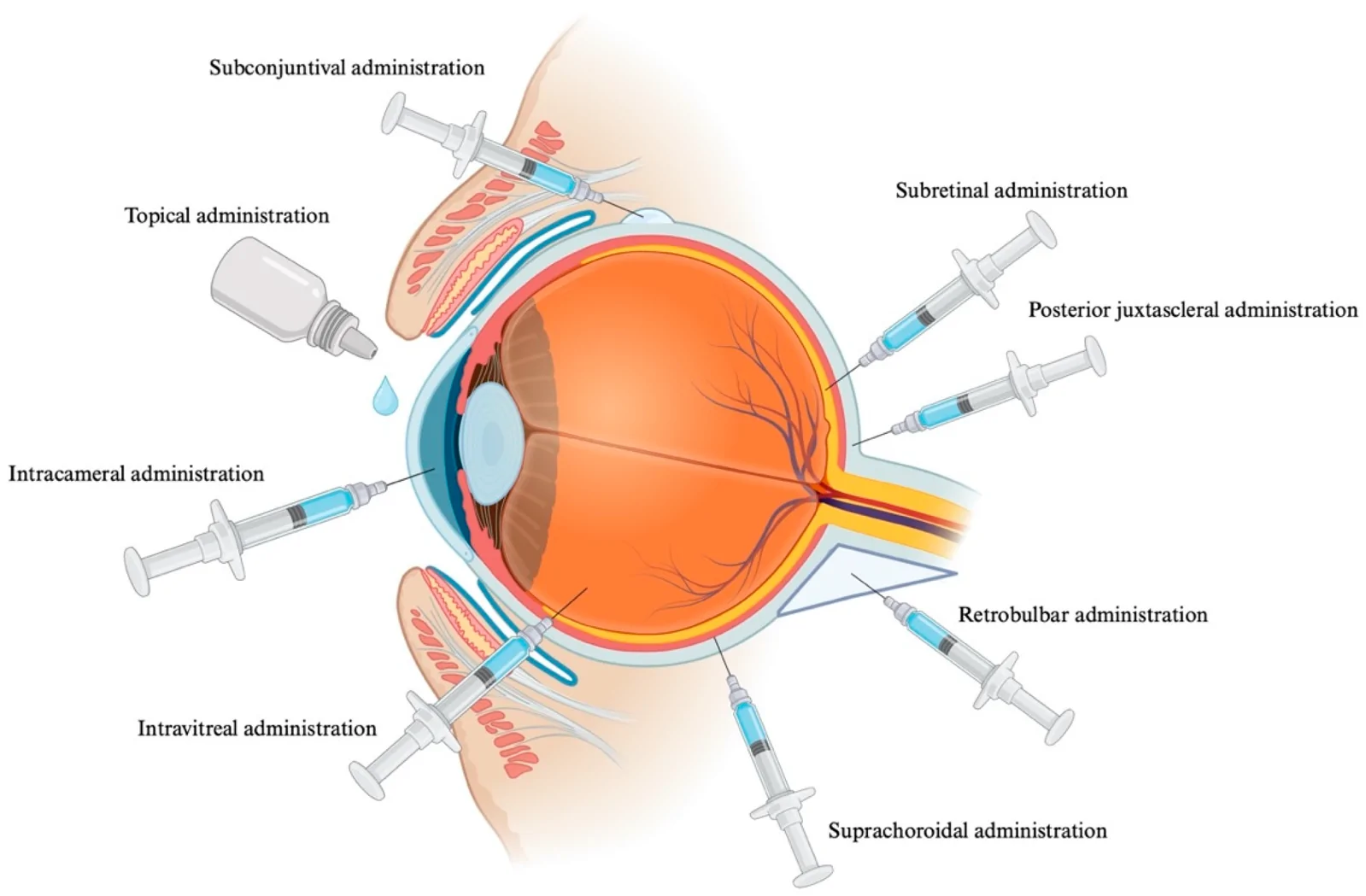
Routes of ophthalmic drug administration, including administration to the anterior segment and the posterior segment. Created in BioRender. Otero, F. (2026) https://core.local.biorender.dev/api/short-link/6bfyvdq.

Ophthalmic administration (Figure 6) is challenging due to the complexity of the anatomy and physiology of the eyeball, where traditional methods of ocular therapy present some drawbacks depending on the route of administration. Some of these are the poor solubility of the drug in tear fluids, which limits bioavailability; the numerous side effects generated by systemic absorption of the drug due to nasolacrimal drainage; and the need for repeated instillation to ensure an adequate amount of drug on the ocular surface [93].

Administration of HCb via this route would be most appropriate for treating ophthalmic pathologies associated with CblC.

#### 10.3.1. Topical Ophthalmic Administration

Topical ophthalmic administration is the safest, easiest, and least invasive route. This route of administration allows for self-administration, increasing patient adherence to treatment. However, it has significantly low bioavailability (<10%), mainly due to the protective mechanism of the eye, such as nasolacrimal drainage, blinking, and high tear clearance. This route is generally used for the treatment of anterior segment diseases. The formulation used for this route can be microemulsions, hydrogels, etc. [119,120,121].

Topical ophthalmic administration is currently being studied for the treatment of posterior segment eye diseases. Yagi et al. conducted a clinical study involving five newborns between 22 and 27 weeks of age with pathological neovascularization in type 2 retinopathy of prematurity, who were treated with dexamethasone eye drops (0.1%). They found that these eye drops suppressed the pathological neovascularization of the disease by modulating mitochondrial activity in the newborns, slowing the progression of the disease [122]. Chastain et al. studied the permeability through the cornea of a 0.1% and 0.3% nepafenac suspension administered topically. A significant therapeutic concentration was observed in the posterior segment of the eye, depending on time and dose. They concluded that in order to obtain a significant concentration in the posterior segment of the eye, high concentrations of the drug are necessary in the anterior segment, favoring the creation of a diffusion gradient through the ocular structures [123]. Davis et al. achieved the delivery of bevacizumab to the retina through topical ophthalmic administration, using liposomes associated with annexin A5 [124].

#### 10.3.2. Intracameral Administration

Intraocular administration consists of injecting the drug directly into the anterior chamber of the eye, with the aim of avoiding the ocular barriers that hinder tropical administration. This route increases the bioavailability of drugs in the anterior chamber, reduces systemic effects and ocular toxicity due to the dilution of the drug in the aqueous humor [125,126].

The main drawbacks of this route are anterior segment toxic syndrome, inflammation after administration, which can cause corneal edema and decreased visual acuity, among other things, as well as toxic endothelial cell destruction syndrome, although the latter is a rare complication caused by the presence of toxic substances in the anterior chamber [127].

Bowers et al. demonstrated the efficacy of an intracameral bimatoprost implant as a treatment for glaucoma, achieving a reduction in intraocular pressure. The use of an implant may also reduce the need for future treatments, as the prolonged release of the drug allows for better control of the disease [128].

#### 10.3.3. Intravitreal Administration

Intravitreal administration is the most common method used to treat diseases of the posterior segment of the eye. It involves injecting the drug directly into the vitreous humor. Direct administration into the vitreous humor maintains drug levels and avoids the blood–retinal barrier. The main drawbacks are that it is an invasive route that can cause retinal detachment or hemorrhage, which makes it difficult for the patient. In addition, repeated intravitreal injections can cause damage to the ocular surface and lead to other pathologies, such as dry eye syndrome [129]. This route is used for the selective administration of molecules such as proteins or peptides, among others [120,130].

Neovascularization associated with AMD is one of the most common conditions treated with intravitreal injections of ranibizumab, a monoclonal antibody fragment that inhibits VEGF. Clinical studies by Brown et al. and Rosenfeld et al. demonstrated for the first time that a significant number of patients experienced improvements in vision after completing treatment. This has led to intravitreal injection of anti-VEGF agent becoming the first-line treatment for AMD [131,132,133]. Park et al. conducted a phase I study evaluating the intravitreal administration of autologous CD34+ stem cells from bone marrow. The results showed that it is a well-tolerated treatment in eyes with vision loss due to retinitis pigmentosa [134].

#### 10.3.4. Periocular Administration

##### Subconjunctival Administration

Subconjunctival administration is an alternative route to intravitreal injection for the administration of drugs in the treatment of retinal diseases [135]. It is considered a less invasive technique that offers the possibility of administering larger volumes compared to the intravitreal route of administration [136]. It also reduces side effects. The drug is injected under the conjunctival membrane covering the sclera, bypassing the conjunctival epithelial barrier and creating a reservoir that releases the drug in a controlled manner [120]. This provides direct access via the transscleral route [66], increasing its bioavailability in the aqueous humor compared to the topical route, which is impeded by the corneal barrier.

However, the bioavailability of drugs administered subconjunctivally is often limited due to their extensive absorption by the lymphatic and blood circulatory systems rather than their intraocular distribution. As a result, frequent injections are required, which may carry risks associated with administration, such as conjunctival edema and subconjunctival hemorrhage [136]. In addition, treatments administered by this route can cause interference in the visual field [137]. Shinde et al. developed a biodegradable PLGA (poly(lactic-co-glycolic acid) microparticle loaded with etoposide and carboplatin for subconjunctival administration in the treatment of retinoblastoma [138].

##### Retrobulbar Administration

Retrobulbar administration is considered an alternative route to intravitreal administration in the pharmacological treatment of posterior eye diseases involving the topic nerve or retrobulbar spaces [125]. The main disadvantages associated with this route of administration are retrobulbar hemorrhages and damage to the optic nerve [66].

Lincoff et al. described a choroidal concertation of IFN-2 higher than the serum concentration after retrobulbar injection. This suggests this route of administration as an option for the treatment of choroidal neovascularization [139]. In addition, Thach et al. conducted a retrospective study highlighting the validity of the retrobulbar route for the administration of corticosteroids in the treatment of cystoid macular edema persistent to treatment with topical drugs [140].

#### 10.3.5. Posterior Juxtaescleral Administration

Posterior juxtaescleral administration is a route through which administration close to the macula is achieved. Its main advantage is increased drug penetration due to the reduced thickness of the sclera at this point [141]. This route also allows the administration of depot formulations, maintaining constant drug levels in the macula for up to 6 months [120].

Augustin et al. conducted a clinical trial using an anecortave acetate depot administered in the macular area through a curved cannula for the treatment of AMD, thus avoiding perforation of the eyeball. This study obtained positive results in maintaining vision and reducing the growth of choroidal neovascularization in patients with age-related macular degeneration [142].

#### 10.3.6. Suprachoroidal Administration

The suprachoroidal space does not interfere with the optic pathways, making it a good route for treating diseases related to the outer retina, photoreceptors, RPE, and choroid. Compared to other periocular routes of administration, drug diffusion from the suprachoroidal space avoids several ocular barriers. The suprachoroidal space could act as a potential reservoir within the eye, and sustained-release formulations or devices could optimize diffusion kinetics from this space. Finally, this space provides a safer route for larger biological and/or immunogenic agents. It also has some disadvantages, such as suprachoroidal hemorrhage and choroidal detachment [66].

Choi et al. evaluated the use of hydrogel-based microneedles to deliver drugs into the space between the sclera and the choroid of the eye. The biodegradable nature of the materials has made it possible for the drug to be released from the hydrogel matrix as it degrades. This allows for effective diffusion of the drug into the suprachoroidal space throughout the eye, reducing the risk associated with the use of non-biodegradable materials and repeated administration [143]. Yeh et al. conducted a phase 3 clinical trial for the treatment of macular edema with triamcinolone acetonide, which improved vision in 47% of patients without the increase in intraocular pressure that occurs with topical, periocular, and intravitreal corticosteroid treatment [144].

#### 10.3.7. Subretinal Administration

Subretinal administration involves delivering the drug directly into the subretinal space, located between the RPE cells and the photoreceptors, using a precise and minimally invasive injection [66].

Marmor et al. highlighted that elimination of macromolecules from subretinal space is very slow after subretinal administration. This suggests that this route is a good option for increasing the local bioavailability of macromolecules [145].

CblC is a chronic disease, so a comfortable and painless topical ophthalmic administration is required. The topical ophthalmic administration provides these key advantages, ensuring patient acceptance; however, it presents the challenge of delivering the drug to the posterior segment of the eye. Promising advanced delivery systems are currently being developed that would enhance the delivery and bioavailability of HCb to the posterior segment of the eye (see Section 10.4). For example, the use of nanometric systems that would enable the delivery of HCb to the posterior segment of the eye. The inclusion of these systems is increasing the possibilities of choosing this type of administration as a key treatment for this disease.

The main disadvantage of other administration methods is the need for recurring injections in chronic diseases. In the same way, advanced delivery systems (see Section 10.4) could be used to reduce the number of injections required, thereby also reducing the associated adverse effects. For example, the use of a controlled-release intravitreal or intracameral implant loaded with HCb would maintain a constant and high concentration of the drug in the posterior segment of the eye.

### 10.4. Systems for Improving the Bioavailability of HCb via the Ocular Surface

**Figure 7:**
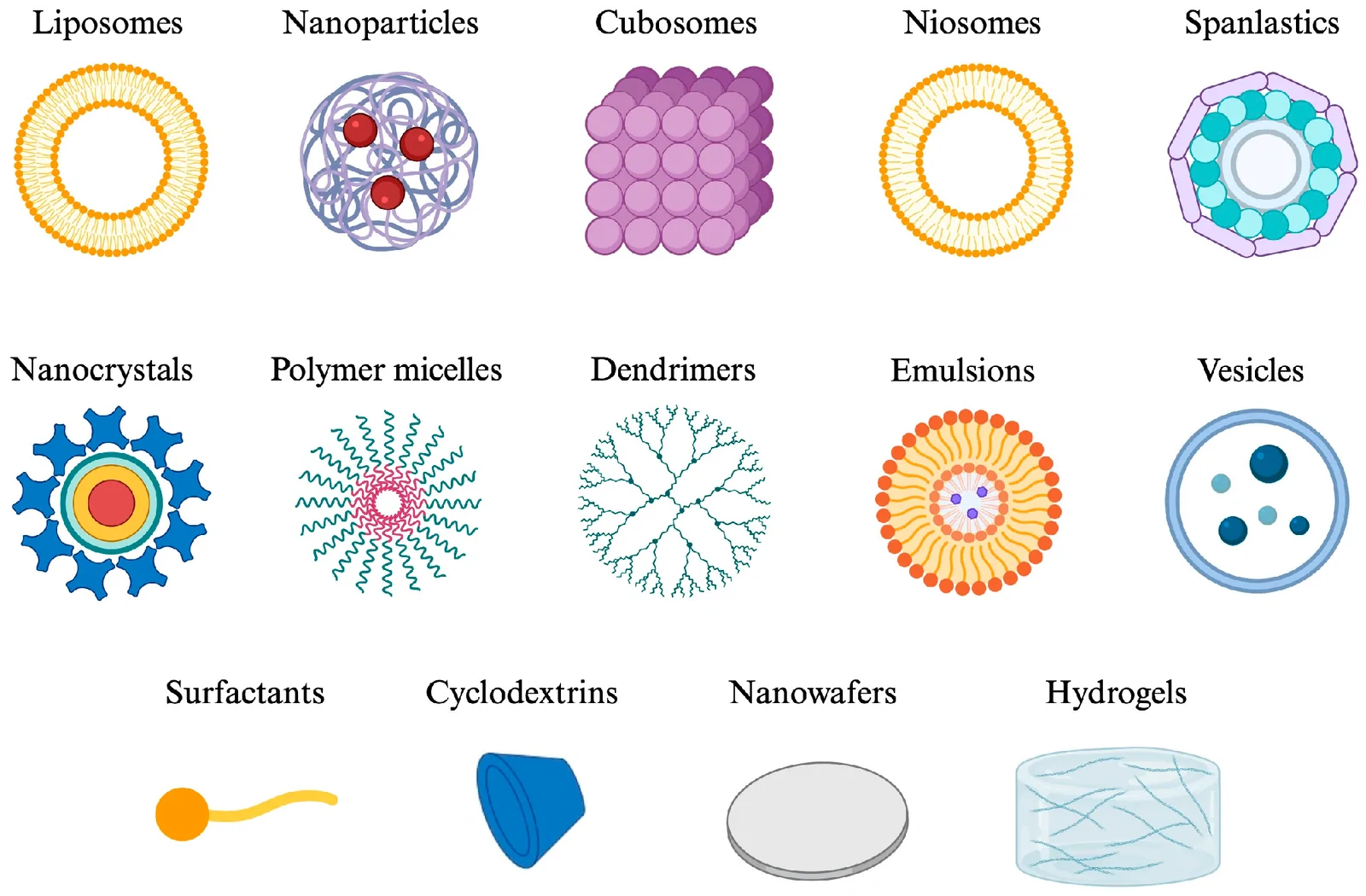
Advanced delivery systems to enhance the permeability, bioavailability and therapeutic efficacy of HCb. Table 4 summarizes the information regarding the characteristics of these systems. Created in BioRender. Otero, F. (2026) https://core.local.biorender.dev/api/short-link/bp2cibc.

CblC homocystinuria is more prevalent in neonatal and pediatric patients. Consequently, the most appropriate route of administration is topical ophthalmic application, with the aim of minimizing the distress and invasiveness of treatment in this particularly vulnerable population. However, this route of administration presents numerous anatomical and physiological barriers that limit the absorption of the active ingredient; there is particular interest in the use of complex and innovative delivery systems capable of increasing the permeability, bioavailability and therapeutic efficacy of HCb in the treatment of CblC (Figure 7). Furthermore, HCb and other Cbl derivatives share certain similar characteristics, such as size and hydrophilicity/lipophilicity balance, which may limit incorporation into certain systems, and which must be taken into account when choosing between the different options (Table 3).

**Table 3:** Physicochemical properties of Cbl derivatives and suitable systems based on these properties [136,146,147].

Characteristic	Cbl Derivatives	Implication for Ocular Delivery	Potentially More-Suitable Platforms
Molecular size	High, ≈1.35 kDa	Limits passive diffusion across the cornea and other ocular barriers	Microneedles, nanocarriers, CDs [136,146,147]
Water solubility	Favorable	Facilitates the use of aqueous formulations	Hydrogels, surfactants, CDs, contact lenses [146]
Affinity for lipid phases	Low	May hinder direct incorporation into lipid matrices and permeation across lipophilic membranes	Liposomes; nanoparticles containing aqueous compartments [136,146,147]
Structural complexity	High: corrinoid structure with numerous functional groups	May complicate chemical modifications without altering biological activity	Prodrugs/conjugates require careful design and characterization [136]

#### 10.4.1. Surfactants/Stabilizers

Surfactants (or surface-active agents), whether natural or synthetic, act by reducing surface tension at an interface. They act as stabilizers for emulsions and suspensions, improving drug stability and, consequently, bioavailability, due to their amphiphilic nature, with a polar head and non-polar tail. Surfactants affect the physiochemistry of the system and the biocompatibility of formulations, and may be anionic, cationic, amphoteric or non-ionic. Non-ionic surfactants, such as sorbitan esters and polysorbates, are the preferred surfactants in ocular nanocarriers due to their low toxicity. Cationic surfactants exhibit greater toxicity, as they interact with the negative charges present in biological molecules, such as DNA or cell membranes [148]. Some surfactants commonly used in ophthalmology are: poloxamers, Cremophor RH 60, tiloxapol, Kolliphor, Transcutol HP and vitamin E TPGS (polyethylene D-alpha-tocopherol glycol 1000 succinate) [149,150]. Due to their chemical structure, surfactants act as permeation enhancers at the level of the corneal epithelium by inhibiting the P-glycoprotein efflux pump and disrupting the tight junctions between epithelial cells, thereby facilitating transcellular corneal permeation [151,152].

The presence of surfactants affects the final particle size and stability, thereby influencing the bioavailability and release of the active ingredients via this route. Furthermore, surfactants can interact with the active ingredients present, increasing their solubility and reducing their premature degradation [153]. At high concentrations and with prolonged use, they may cause corneal irritation, although this is not usually a problem, as the concentration chosen is typically the minimum effective level required to stabilize the formulation without causing damage [151,154].

#### 10.4.2. Polymeric Micelles

Polymeric micelles are formed through the self-assembly of amphiphilic polymers when the critical micelle concentration (CMC) is exceeded. When the CMC is exceeded, the hydrophobic tail of the polymer assembles to form a hydrophobic core, leaving the hydrophilic head of the polymer on the outer surface [155,156,157]. The internal hydrophobic core can harbor hydrophobic drugs, and the external hydrophilic surface increases their solubility in aqueous solutions and their bioavailability. These systems are biocompatible, have low toxicity and increase cellular permeability through the cornea thanks to their deformability and size [158,159].

In addition to these advantages, polymeric micelles result in clear solutions that do not impair vision when administered. Some types possess mucoadhesive properties that increase corneal retention time, protecting the drug from degradation and allowing for the modification of drug release. Among the disadvantages of these systems is the fact that tearing can dilute the formulation, preventing it from reaching CMC. Furthermore, they have a limited drug-loading capacity, particularly large molecules, and limited chemical and physical stability, leading, for example, to aggregation of phenomena [160,161].

El-Shashed et al. developed a hydrogel based on polymeric micelles containing fexofenadine for the treatment of allergic conjunctivitis. They achieved an encapsulation efficiency between 60 and 90% and faster recovery in in vivo trials [162], whilst Jaiswal et al. developed poloxamer 407 (P407) polymeric micelles containing itraconazole for the treatment of fungal keratitis. These systems had a mean particle size of 79.99, an encapsulation efficiency of 91% and increased transcorneal penetration compared with the commercial eye drop Itral^®^ [163].

#### 10.4.3. Cyclodextrins (CDs)

CDs are toroidal cyclic oligosaccharides capable of housing various molecules within their structure, forming inclusion complexes. They have a hydrophilic outer surface and a lipophilic inner cavity, allowing both hydrophilic and lipophilic molecules to bind to them. In aqueous solution, a dynamic equilibrium is established between the inclusion complex, the free CD molecules and the free drug [164].

There are different types of CD classified according to the number of a-D-glucopyranose units linked by alpha-D-(1,4) bonds: alpha-cyclodextrin (α-CD), beta-cyclodextrin (βCD), gamma-cyclodextrin (ɣCD) and synthetic CDs derived from natural ones, such as 2-hydroxypropyl-β-cyclodextrin (HPβCD) or hydroxypropyl-ɣ-cyclodextrin (HPɣCD), amongst others [165,166]. Synthetic CDs have greater water solubility compared to natural CDs [167].

CDs are used because of their ability to form inclusion complexes with drugs, thereby improving their water solubility, enhancing their stability, reducing toxicity and improving transcorneal penetration [168,169,170]. The disadvantages stem from the use of high concentrations of CDs, which may interact with physiological molecules such as cholesterol, an essential component of cell membranes. Furthermore, they may also have limitations regarding drug release, depending on whether the complexation equilibrium is shifted in favor of the drug–CD complex or the free drug [171,172].

Klahan et al. developed a tropical ophthalmic formulation of fenofibrate by forming polypseudorotaxanes with HPβCD, P407 and Soluplus^®^, for the treatment of AMD and diabetic retinopathy. A penetration study in porcine cornea revealed an increase in the passage of fenofibrate through the sclera [173]. Huang et al. developed ophthalmic drops containing CD to deliver a VEGF inhibitor for the treatment of AMD. In vivo studies demonstrated excellent biodistribution in the choroid and retina, with results comparable to intravitreal injections of the same drug [174].

Currently there are several medicines and medical devices on the market that contain cyclodextrin: Clear eyes^®^ with βCD, Bilaxten^®^/Ibis^®^ with HPβCD and Voltaren^®^/Voltadol^®^ with HPɣCD.

#### 10.4.4. Hydrogels

Hydrogels are systems formed by three-dimensional polymer chains, with a high capacity to absorb water and maintain their structure [175,176]. They are biocompatible and biodegradable systems, capable of retaining drugs within the cross-linked matrix, thereby potentially increasing the residence time of the active ingredient on the ocular surface [177]. Furthermore, the swelling of the hydrogel acts as a protective mechanism for the drug against premature degradation within the body [177,178]. Depending on the polymer or mixture of polymers that form part of the hydrogel composition, it will exhibit different gelation properties.

The benefits associated with the use of hydrogels include increased contact time between the drug and the ocular surface, controlled release, enhanced comfort and mechanical protection of the cornea, preventing irritation and facilitating the healing of corneal micro-lesion. Furthermore, the use of hydrogels that form in situ on the ocular surface allows for administration in liquid drops, thereby improving ease of administration. Instead, the main disadvantages of hydrogels are related to blurred vision, difficulty in sterilizing them using conventional methods (particularly with thermolabile polymers), the potential for interactions with active substances that reduce their bioavailability and limited physicochemical stability [179,180,181].

Mohan et al. developed three pH-dependent, temperature-dependent and ion-dependent ciprofloxacin hydrogels, using Carbopol 940, P407 and gellan gum respectively. For all formulations, they achieved good antibacterial activity, a good release profile, increased retention on the ocular surface and formulation stability, compared with commercial products [182]. Numerous hydrogels have already been approved by the FDA, such as ReSure^®^, a polyethylene glycol hydrogel approved by the FDA for the sealing of corneal incisions [183]. Another example is Thealoz Duo Gel eye drops, a hyaluronic acid and trehalose hydrogel for the treatment of severe dry eye [184].

##### Mucoadhesive Hydrogels

Mucoadhesive hydrogels consist of polymer solutions capable of interacting with the acid residues (sialic acid residues) of mucin present in the tear fluid, thereby increasing the duration of contact with the ocular surface [185]. Numerous polymeric solutions are currently used to increase mucoadhesion at the topical–ophthalmic level, such as methylcellulose, hydroxyethylcellulsoe, carboxymethylcellulose, polyvinyl alcohol, or hyaluronic acid [119,186].

##### In Situ Gelling Hydrogels

In situ gelling hydrogels are liquid polymeric systems that cross-link in the presence of a stimulus or a combination of stimuli, such as the temperature of the ocular surface, pH, or the presence of certain ions in tears (Na^+^, K^+^, Mg^2+^, Ca^2+^). They can be classified as thermosensitive, pH-sensitive, and ion-sensitive in situ gelling systems. The main advantages they offer are increased corneal retention, precise dose delivery and controlled drug release [187]. Some polymers, such as poloxamer 407, carboxymethylcellulose or collagen, exhibit temperature-sensitive gelation [188,189]. Others, such as chitosan, carbomers or methacrylic acid, exhibit pH-dependent gelation [190,191], whilst others, such as alginate, gellan gum or carrageenan, exhibit ionic gelation [192,193,194].

#### 10.4.5. Liposomes

Liposomes are vesicular systems ranging in size from 20 nm to 1 µm, consisting of a phospholipid bilayer and an aqueous core. They are classified structurally according to the number of lipid bilayers. They possess numerous characteristics, such as biocompatibility, biodegradability and the ability to encapsulate both hydrophilic and lipophilic drugs, as well as those with high molecular weights [195,196]. Furthermore, the use of amphiphilic lipids allows for greater interaction with tear components, promoting the retention of the formulation on the ocular surface. This system reduces drug toxicity, prolongs the half-life of drugs and improves the absorption of active ingredients through the ocular barriers [197,198]. On the other hand, liposomes are systems prone to oxidation, have limited stability in aqueous systems, may aggregate, thereby reducing the uniformity of the system, and, with certain types of molecules, have low encapsulation efficiency [199,200].

Nowadays, the use of these systems to enhance transcorneal penetration is on the rise. Shen et al. developed ganciclovir liposomes, whilst Hosny developed a liposomal hydrogel containing ciprofloxacin and carbopol. In both cases, the formulation increased transcorneal penetration compared with the free drug solution [201,202]. Meanwhile, Chen et al. developed cationic liposomes containing tacrolimus for the treatment of dry eye, demonstrating benefits in reducing intracellular ROS and inflammatory factors associated with the condition [203]. Currently, there is a medical device on the market for this condition that includes liposomes in its composition (Aquoral^®^ Lipo, Navilipo^®^).

#### 10.4.6. Nanoparticles

Nanoparticles are tiny particles ranging in size from 1 to 1000 nm, depending on their constituent material and the production technique used. They generally consist of a homogeneous matrix or a core, an outer layer, and a surface layer, which may incorporate surfactants, metal ions or polymers [204]. They can be composed of various substances, such as lipids, inorganic substances like graphene or gold, and polymers, amongst others [205]. They exhibit a wide variety of shapes and structural configurations, which depend both on the material they are made of and the production technique. For example, they may exhibit cylindrical tubular, conical, hollow-core, spiral or flat morphologies, amongst others. These geometries significantly influence their physiochemical properties, functionality and potential applications in various fields [206].

The main advantages of nanoparticles are increased absorption and retention, protection of the drug against degradation, and the potential for targeted delivery through surface modifications. Furthermore, they are compatible with various ophthalmic delivery systems (eye drops, inserts, medicated lenses, etc.). The main disadvantages are related to the limited stability of these systems, as aggregation may occur, sterilization presents difficulties, and some types of nanoparticles may accumulate in the nasolacrimal ducts, causing variability in release and bioavailability. Furthermore, there are still uncertainties regarding regulatory standards or conditions for nanoparticles intended for ophthalmic use, and the costs associated with the industrial production of such systems are very high [99,207].

##### Polymeric Nanoparticles

Polymeric nanoparticles are systems composed of polymers; the most used are biodegradable polymers, such as PLGA, PLA, PCL, chitosan or alginate. Polymeric nanoparticles offer many advantages in the delivery of drugs for the treatment of ophthalmic conditions, due to their bioadhesive properties. Bioadhesion is important for increasing the contact time of the formulation with the ocular surface, as otherwise the nanoparticles are cleared via the nasolacrimal drainage system [208].

Beirampour et al. developed PLGA and PCL (poly-e-carprolactone) polymeric nanoparticles loaded with baricitinib for the treatment of uveitis and dry eye. They observed an increase in corneal permeability and the amount of drug retained in the cornea, achieving a depot effect [209]. Hibrahim et al. developed PLGA, PCL and PLA (poly(l-lactide)) polymeric nanoparticles loaded with celecoxib for the treatment of the diabetic retinopathy and AMD, achieving encapsulation efficiencies of 97% [210].

##### Solid Lipid Nanoparticles (SLNs)

SLNs were developed as an alternative to traditional carriers, such as liposomes and polymeric nanoparticles. They consist of one or one more biodegradable and biocompatible lipids that are solid at room temperature, and a surfactant that stabilizes their matrix, into which both fat-soluble and water-soluble drugs can be incorporated. These systems have limited stability over time, as they can lead to drug loss from the matrix and lipid oxidation [152].

Attama et al. developed SLNs containing diclofenac for tropical ophthalmic administration, obtaining a formulation with high encapsulation capacity (over 90%) and capable of penetrating the cornea [211]. Nair et al. developed SLNs loaded with clarithromycin for the treatment of bacterial ophthalmic infections, achieving increased corneal penetration compared with the control clarithromycin solution [212].

##### Nanostructured Lipid Carriers (NLCs)

NLCs are second-generation lipid systems composed of a mixture of lipids. They can incorporate both lipid-soluble and water-soluble drugs and minimize drug leaching from the lipid matrix during storage, thereby increasing stability. Like other nanoparticles, they help reduce toxic effects and increase bioavailability and permeation [213,214].

Velasco et al. developed NLCs containing adalimumab for the topical ophthalmic treatment of retinitis pigmentosa, successfully overcoming the corneal barriers and exerting an effect at the retinal level [215]. Kumar et al. developed NLCs with itraconazole for the topical ophthalmic treatment of fungal infections, demonstrating an increase in the zone of inhibition in antifungal assays compared with the commercial formulation [216].

##### Hybrid Lipid–Polymer Nanoparticle (PLN)

PLNs are emerging as highly promising candidates for the delivery of ophthalmic drugs due to their tunable physicochemical properties and their ability to enhance drug uptake and retention in the eye [217]. These nanoparticles consist of a lipid layer, mainly phospholipids, and a polymeric core; biodegradable polymers such as PEG or PLGA are often used [218]. These systems offer improved stability, controlled release and the ability to encapsulate both hydrophilic and lipophilic drugs. Furthermore, the lipid layer bears a resemblance to the cell membrane, facilitating penetration through cell membranes [217,218]. The disadvantages of these systems typically include phagocytic uptake, potential immune reactions, uncontrolled distribution to tissues and a short half-life [219].

Kaviarasi et al. investigated hybrid particles for delivering difluprednate for the treatment of uveitis, achieving increased corneal penetration compared with the drug solution [220], and Liu et al. developed PLNs containing moxifloxacin hydrochloride. These nanoparticles increased penetration and residence time to the corneal surface, as the hyaluronic acid in the PLN accelerated uptake via endocytosis [221].

##### Niosomes

Niosomes are vesicular systems formed by the assembly of a non-ionic surfactant with cholesterol in an aqueous medium, resulting in bi- or multi-layered nanoparticles [222]. The use of niosomes began in the cosmetic industry, although it is now expanding into therapeutics. This system improves the bioavailability and encapsulation of drugs, as its lamellar and flexible structure allows for the encapsulation of both hydrophilic and lipophilic drugs, protecting the drug from rapid clearance and degradation. Furthermore, the vesicular structure allows for better permeation through biological tissues [222,223]. On the other hand, the loading capacity for highly hydrophilic drugs may be limited, hindering the drug’s reach to the posterior regions of the eye when applied topically and potentially requiring controlled storage conditions [222].

Abdelbary et al., for example, developed gentamicin niosomes for the treatment of bacterial eye infections [224]. Allam et al. developed niosomes containing betaxolol incorporated into an in situ-forming gel for the treatment of glaucoma, thereby increasing the drug’s bioavailability [225]. Gugleva et al. developed doxycycline niosomes for the topical treatment of ocular conditions involving metalloproteinase 0, such as rosacea, which causes dry eye syndrome and corneal ulcerations [226].

#### 10.4.7. Dendrimers

Dendrimers are globular nanostructured polymers with reactive end groups, which may carry negative, positive or neutral charges. Thanks to these functional groups, they can target any part of the body, directing the drug to the site of action [227]. Such systems can encapsulate hydrophobic drugs within their internal cavities, increasing their solubility in water, or conjugate them to the surface of the dendrimer. Dendrimers present toxicity issues related to surface charges. For example, cationic dendrimers are more toxic due to the interaction of their positive charge with biological negative charge [228,229]. On the other hand, ophthalmic formulation containing dendrimers are being designed that show no signs of toxicity or irritation. Shaunak and al. linked two dendrimers and discovered that their combined action exhibited immunomodulatory properties, preventing the formation of scar tissue [230].

Iezzi et al. developed PAMAM (hydroxyl-terminated polyamidoamine) dendrimers conjugated with fluocinolone acetonide for the intravitreal treatment of neuroinflammation in retinal degeneration. The dendrimers developed were able to halt retinal degeneration, yielding satisfactory results with this method of administration [231]. Mishra et al. studied a dendrimer-based formulation for the delivery of acetazolamide for the treatment of glaucoma in rabbits, achieving a sustained and prolonged reduction in intraocular pressure [232].

#### 10.4.8. Nanocrystals

Nanocrystals are particles of pure drug, with particle sizes ranging from 1 to 1000 nm, stabilized with surfactants or polymeric solutions [233,234]. This system is widely used to increase the solubility of drugs that are poorly soluble in water; furthermore, it can increase the active ingredient load and improve adhesion and penetration through mucous membranes due to its nanometric size, which facilitates absorption through biological membranes [235,236]. Their use in ophthalmic applications is on the rise due to their lower toxicity and greater ocular absorption compared to other nanometric systems [234]. The main disadvantage of nanocrystals is that they have limited physical stability, as they can give rise to aggregation or coalescence phenomena. To prevent this, it is necessary to use stabilizers such as surfactants or polymeric solutions like poloxamers [234].

Tuomela et al. developed brinzolamide nanocrystals, achieving satisfactory results in reducing intraocular pressure in in vivo models [237]. Tetyczka et al. developed itraconazole nanocrystals for direct printing onto commercial soft hydrogel contact lenses, demonstrating a promising method of application for these systems [238]. Loi et al. developed a suspension of diclofenac nanocrystals for the treatment of corneal inflammatory conditions, achieving greater corneal penetration compared with the commercial formulation [239].

#### 10.4.9. Cubosomes

Cubosomes are systems formed by the dispersion of amphiphilic lipid molecules in a liquid crystalline phase, with a honeycomb or cubic structure [240]. They range in size from 100 to 500 nm, have a high loading capacity and can carry both hydrophilic and lipophilic drugs [241,242].

These are promising systems due to their thermodynamic stability and the ability to control drug release over time. Furthermore, these systems are biocompatible, bioadhesive and improve transcorneal penetration [242,243]. On the other hand, cubosomes are subject to physicochemical instability, which may be exacerbated by certain sterilization techniques that can compromise their structure, potentially resulting in opaque formulations that reduce visual comfort. Furthermore, they are difficult to scale up, and at present, there are still few studies available on their application in the ophthalmic field, although some authors describe these systems for the treatment of ophthalmic conditions [244].

Teba et al. developed cubosomes loaded with acetazolamide as a treatment for glaucoma, improving the drug’s solubility and transcorneal permeability [243]. Bhageerathy et al. evaluated the potential use of moxifloxacin hydrochloride cubosomes for the treatment of bacterial conjunctivitis. They obtained promising results in terms of safety, sustained release and transcorneal permeability of the drug [245].

#### 10.4.10. Emulsions

Emulsions are uniform dispersed systems consisting of two or more immiscible liquids stabilized by a surfactant. When the two phase are mixed, an outer or continuous phase and an inner or discontinuous phase are formed [246,247]. There are three main types of emulsion: water-in-oil (W/O), oil-in-water (O/W) and double emulsion, namely water-in-oil-in-water (W/O/W) and oil-in-water-in-oil (O/W/O) [247]. Emulsions can encapsulate both lipophilic and hydrophilic drugs, improving drug permeability and bioavailability by increasing the solubility of poorly soluble medicines [248,249]. Furthermore, emulsions (particularly W/O) have the benefit of protecting the ocular surface and delaying the evaporation of the tear film, thereby increasing the drug’s residence time and reducing the number of daily doses required. On the other hand, they are prone to physical instability (coalescence, aggregation, and creaming). Furthermore, the surfactants used to stabilize them may be irritating to the eye [250].

Yamaguchi et al. developed an oil-in-water (O/W) emulsion containing difluprednate, an anti-inflammatory agent used to treat ocular inflammation following eye surgery [251]. Furthermore, there are already commercially available products formulated as emulsions, such as Restasis^®^. This is an ophthalmic cyclosporine emulsion indicated to increase tear production in patients where this function is suppressed due to ocular inflammation associated with dry keratoconjunctivitis [252].

#### 10.4.11. Spanlastics

Splanlastics are flexible nanocarriers based on non-ionic surfactants and edge activators, which enhance the deformability of lipid bilayers and act as destabilizers of vesicular membranes, leading to increased absorption through biological membranes. They are non-immunogenic, biodegradable, and safe. They also protect the active ingredient from degradation and can have a functionalized surface, for example with pegylated molecules or peptides, improving drug delivery [253]. The main disadvantages stem from the complexity and cost of production, their physical instability, and the fact that they cannot withstand autoclave sterilization [254].

Kakkar and Kaur developed nanoplastics containing ketoconazole for topical ophthalmic use, evaluating corneal permeability in comparison with a niosomal formulation. The nanoplastics improved penetration into the posterior segment of the eye, demonstrating that this type of carrier is effective for delivering drugs to the posterior segment of the eye [255].

#### 10.4.12. Vesicles

Extracellular vesicles are lipid-rich vesicles released by cells, containing proteins, lipids, and nucleic acids. They can act locally or systemically, transporting their contents to target cells. Notable among extracellular vesicles are exosomes, ranging in size from 30 to 150 nm, derived from the cellular endosomal apparatus, and macrovesicles, ranging from 150 to 1000 nm, which originate from the plasma membrane. These systems offer advantages such as protection of the drug against degradation, controlled release, and the ability to transport molecules to cells via endocytosis. They can induce a specific cellular function via a receptor–ligand binding [256,257].

These systems are generally well tolerated and exhibit antioxidant and anti-apoptotic effects in retinal and corneal cells. However, their load-carrying capacity is limited, and aggregation and degradation may occur over time. At an industrial level, their scalability is challenging, as large-scale production is complex and costly; at a regulatory level, further information is required regarding dosage, biodistribution and long-term effects [258,259].

Chopra et al. conducted a study on the use of exosomes in dry eye syndrome, demonstrating that their use leads to improved tear production, reduced lymphocyte infiltration, greater structural integrity, and decreased apoptosis of epithelial cells [260]. Liu et al. developed a formulation of platelet-derived extracellular vesicles loaded with kaempferol to treat corneal neovascularization. Following topical application in in vivo trails in mouse eyes, they demonstrated the anti-angiogenic and anti-inflammatory activity of the formulation [261].

#### 10.4.13. Nanowafers

Nanowafers are transparent, nanoscale membranes or drug-loaded discs that can be easily applied to the surface of the eye. They are composed of biodegradable and biocompatible polymers that degrade and are easily eliminated over time. Nanowafers act as drug reservoirs, controlling drug release and increasing retention time on the ocular surface, thereby facilitating drug absorption into the ocular tissues [149]. They exhibit good long-term stability and good adhesion to the ocular surface. On the other hand, their presence may cause irritation and a foreign body sensation in the eye. Finally, nanowafers are difficult to produce and can carry only a limited amount of drug [262].

Marcano et al. developed a system to overcome the problems of multiple daily doses (6–12 times a day) and the side effects associated with cysteamine in the treatment of corneal cystinosis (a rare metabolic disorder) by producing biodegradable cysteamine nanowafers. The results of the study demonstrated increased therapeutic efficacy at low concentrations and with a single daily dose, as well as improved safety profile [263].

**Table 4:** Summary of the main advantages and disadvantages of advanced delivery systems.

	Advantages	Disadvantages
Surfactants/ stabilizers	Increase drug stability, solubility and bioavailability [148] Decrease drug degradation [148] Increase drug permeability [151,152]	Cationic surfactants exhibit greater toxicity [148] Ocular irritation at high doses [151,154]
Polymeric micelles	Increase drug permeability and bioavailability [158,159] Biocompatibility and mucoadhesiveness [158,159] Decrease drug degradation [160,161]	The formulation can be diluted, preventing it from reaching the CMC [160,161] Limited load capacity [160,161] Limited stability [160,161]
Cyclodextrins	Increase drug permeability and bioavailability [168,169,170] Decrease toxicity [168,169,170]	Interaction with membrane components at high concentrations [171,172] Drug release dependent on the complexation equilibrium [171,172]
Hydrogels	Biocompatibility and biodegradability [179,180,181] Mucoadhesiveness [179,180,181] Decrease drug degradation [177,178] Control release [179,180,181]	Blurred vision [179,180,181] Difficult sterilization [179,180,181] Limited stability [179,180,181]
Liposomes	Biocompatibility and biodegradability [195,196] Decrease drug toxicity [197,198] Increase in the half-life of drugs [197,198]	Limited stability [199,200] Limited load capacity [199,200] Oxidation and aggregation phenomena [199,200]
Nanoparticles	Increase drug permeability and bioavailability [99,152,207] Decrease drug degradation [99,207,213,214] Targeted delivery [99,207]	Limited stability [99,207] Difficult sterilization and high prices [99,207] Accumulation in the nasolacrimal ducts [99,207,208] Regulatory issues [99,207]
Dendrimers	Targeted delivery [227] Increase drug solubility [228,229]	Tissue toxicity [228,229]
Nanocrystals	Increase drug solubility [235,236] Increased adhesion and permeability in biological membranes [234,235,236]	Limited stability [234]
Cubosomes	Thermodynamic stability [242,243] Control release [242,243] Biocompatibility and mucoadhesiveness [242,243,244] Increase drug permeability [242,243]	Limited physicochemical stability [244] Difficult sterilization [244] Impair vision [244] Large-scale industrial complex [244]
Emulsions	Increase drug solubility, permeability and bioavailability [248,249] Protect the surface of the eye [248,249] Decrease tear evaporation [248,249]	Limited stability [250] Toxicity associated with the surfactants used [250]
Spanlastics	Increase drug permeability [253] Biocompatibility [253] Decrease drug degradation [253] Targeted delivery [253]	Large-scale industrial complex [254] Difficult sterilization and high prices [254] Limited stability [254]
Vesicles	Decrease drug degradation [256,257] Control release [256,257] Biocompatibility [258,259]	Limited stability [258,259] Limited load capacity [258,259] Large-scale industrial complex [258,259] Difficult sterilization and high prices [258,259] Regulatory issues [258,259]
Nanowafers	Biocompatibility and biodegradability [262] Control release [149] Increased residence time of the drug in the eye [149] Good stability and ocular bioadhesion [262]	Large-scale industrial complex [149,262] Limited load capacity [149,262] Irritation [262] Foreign body sensation [262]

## 11. Opinion

CblC homocystinuria is a highly prevalent condition in children [7]. It is therefore essential to choose a method of treatment administration that is convenient and minimally invasive. In this review, we focus on evaluating the potential for a treatment to half or manage the degenerative eye conditions associated with CblC homocystinuria. Among the various routes of administration previously described for the delivery of drugs to the eye, the topical route represents the most convenient option, as it promotes treatment adherence compared to other methods such as intravitreal injections [121].

The eye is an immunologically privileged organ due to the presence of various barriers, including the cornea, which acts as an additional barrier to the entry of external substances, including drugs. For this reason, it is said that topical ophthalmic administration presents a major barrier or obstacle that must be overcome [65]. It is common for the development of a topical ophthalmic treatment for an eye condition to focus on a single system; however, it is important to bear in mind that combining several of these systems can improve both the retention and the bioavailability of the drug used, thereby improving the treatment of the condition.

In this case, CblC homocystinuria requires a formulation capable of increasing the residence time of HCb on the ocular surface and facilitating the passage of the drug through the cornea. To resolve the issue of HCb passing through the cornea (which is directly influenced by the polarity of HCb), a system is required that is capable of traversing the corneal epithetical layer (the outermost cellular layer of the cornea, which is lipophilic) and that is capable of releasing HCb into the stroma (the middle corneal cell layer, which is hydrophilic). Once HCb has been released into the stroma, it could diffuse more easily through the endothelial layer (the innermost corneal cell layer, which is lipophilic but has wide cell junctions that facilitate the passage of molecules of any nature into the eye). To achieve this, lipid systems with an aqueous core could be used to increase the encapsulation of HCb. Among lipid nanoparticles, NLCs stand out as the best option, as the mixture of solid and liquid confers them a greater drug-loading capacity, prolonged and controlled release, and increased ocular retention [264]. These NCLs can be incorporated into a mucoadhesive system such as a hydrogel, with the aim of increasing the formulation’s retention time on the ocular surface (including the formation of eyelid deposits), thereby increasing the contact time of the NLCs with the ocular surface and allowing their uptake into ocular tissues. Among hydrogels, it could be of interest to use a thermosensitive or ion-sensitive hydrogel. These systems undergo a gel transition once they come into contact with the ocular surface due to physiological temperature or the presence of ions in the tear fluid. Based on this, the formulation would be administered in liquid form, facilitating administration [177,181]. On the other hand, the development of this type of hydrogel to deliver NLCs for topical ophthalmic administration involves, in addition to corneal penetration, the penetration of the HCb through the rest of the ocular structures, such as the conjunctiva. Conjunctival penetration has wider cellular junctions than the cornea, so the passage of molecules (mainly hydrophilic and up to 10 kDa) is greater than through the cornea [265]. Consequently, the use of various advanced delivery systems such as implants, microneedles, inserts and nanoparticles via this route is on the rise, as they result in significant concentrations in the vitreous body and retina [266].

In conclusion, the combination of NLCs with another technology such as mucoadhesive hydrogels is a particularly suitable strategy for the topical ophthalmic administration of HCb, as it addresses the main limitations of this route. Its ability to improve precorneal retention, modulate permeation through the various ocular barriers, and promote controlled drug release makes it an option particularly well-suited to the biopharmaceutical requirements of this molecule. Another potential strategy would be to incorporate NLCs into microneedles for transcorneal or transscleral delivery. This would improve the main limitations of the topical ophthalmic administration, such as nasolacrimal drainage or penetration through the cornea. However, despite its conceptual soundness, the actual efficacy of this type of system in achieving therapeutic levels in the posterior segment following topical administration still requires more robust validation, highlighting the need for preclinical and clinical studies to confirm its translational potential.

## 12. Conclusions

CblC homocystinuria is an autosomal recessive congenital disorder caused by a mutation in the MMADHC gene. This mutation leads to a defect in the conversion of Cbl into its two metabolically active forms, AdoCbl and MeCbl, resulting in multiple systemic disorders.

Diagnosis is based on a combination of biochemical analysis of the blood and urine for the relevant amino acids and organic acids, together with confirmation using molecular biology techniques, primarily PCR. Additionally, prenatal diagnosis can be carried out through amniotic fluid analysis between 16 and 20 weeks of gestation, allowing for early detection in high-risk pregnancies and facilitating early intervention.

From a clinical perspective, this condition is markedly multisystemic in nature and is associated with conditions such as acidosis, congenital heart disease, and anemia, amongst others. In this context, current treatment is based primarily on the IM administration of HCb (Megamilbedoce), supplemented in many cases by adjunctive strategies including betaine, folate, Met and carnitine, with the aim of restoring physiological levels of Hcy and Met, thereby improving overall metabolic control.

However, despite the advances, a significant limitation remains in the management of the ophthalmic manifestation of the disease, which is not adequately addressed by currently available treatments. This therapeutic gap highlights the need to develop specific strategies targeting the ocular system. In this regard, the topical–ophthalmic route represents a highly promising alternative dure to its non-invasive nature, high tolerability, and suitability for the pediatric population. However, the physiological barriers of the eye limit the bioavailability of drugs administrated via this route, which justifies the incorporation of advanced delivery systems to improve drug bioavailability. Systems such as nanoparticles, nanocrystals, and cyclodextrin-based formulations, amongst others, show great potential for improving the ocular penetration and bioavailability of HCb, thereby optimizing its therapeutic efficacy.

Overall, the available data highlights the need to move towards more targeted therapeutic approaches that can effectively address the ophthalmic complications of CblC homocystinuria, making this a priority area of research with clear clinical and translational implications.

## Data Availability

No new data were created or analyzed in this study. Data sharing is not applicable to this article.

## Abbreviations

ABCD4	Subfamily D member 4
AdoCbl	Adenosylcobalamin or cobamamid
AMD	Age-associated macular degeneration
ATP	Adenosine triphosphate
α-CD	alfa-ciclodextrina
BHMT	Betaine-homocysteine methyltransferase
β-CD	beta-ciclodextrina
Ca^2+^	Calcium ion (2+)
Cbl	Vitamin B12 or cobalamin
CblC	Homocystinuria type C
CBS	Cystionin beta-synthase
CD	Cyclodextrina
CSF	Cerebrospinal fluid
CMC	Critical micellar concentration
CN-Cbl	Cyanocobalamin
Co	Cobalt
CSE	Cystationine beta-lyase
DED	dry eye disease
DHF	Dihydrofolate
DNA	deoxyribonucleic acid
Fe	Iron
*γ*-CD	gammaciclodextrina
H+	Hydrogen ion
Hb	Hemoglobin
HC	Haptocorrin
HCb	Hydroxocobalamin
HCO_3_^−^	Ion bicarbonate
Hcy	Homocysteine
HP*β*CD	hidroxipropil-beta-ciclodextrina
HP*γ*CD	hidroxipropil-gamma-ciclodextrina
IDDSs	Implantable drug delivery systems
IF	Intrinsic factor
IFN-2	Interferon-2
IM	Intramuscular
IOP	Intraocular pressure
IV	Intravenous
LMBD1	Membrane receptor domain 1
MA	Methylmalonic acid
MCA	Monochloroacetic acid
MeCbl	Methylcobalamin
Met	Methionine
Me-THF	N5-methyltetrahydrofolate
Mg^2+^	Magnesium ion (2+)
MMA	Methylmalonic aciduria
MMACHC	Methylmalonic aciduria protein type CblC with homocistinuria
MMADHD	Methylmalonic aciduria protein type CblD with homocistinuria
MMC	Methylmalonyl-CoA
MS	Methionine synthase
MTHFR	Methylentetrahydrofolate reductase
MTR	Enzyme methyltransferase
MUT	Enzyme methylmalonyl-CoA mutase
NH_4_^+^	Ammonium ion
NLCs	nanostructured lipid carriers
NMDA	N-mettyl-D-aspartate
PAMAM	Hydroxyl-terminated polyamidoamine
PCL	Poly-e-carprolactone
PCR	Polymerase chain reaction
PLA	Poly(l-lactide)
PLGA	Poly(lactic-co-glycolic acid)
PLN	Hybrid nanoparticles o lipid-polymer nanoparticle
P407	Poloxamer-407
RPE	Retinal pigment epithelium
RTM	Renal thrombotic microangiopathy
ROS	Reactive oxygen species
SAHH	S-adenosyl-L-homocysteine hidrolase
SAH	S-adenosylhomocysteine
SAM	S-adenosimethionine
SC	Subcutaneous
SLN	solid lipid nanoparticles
Suc-CoA	Succinyl-CoA
TC-I	Transcobalamin I o transcobalamin α
TC-II	Transcobalamin II
TDDS	Transdermal drug delivery system
THF	Tetrahydrofolate
TPGS	Polyethylene D-alpha-tocopherol glycol 1000 succinate
VEGF	vascular endothelial growth factor
